# A machine learning framework reveals key drivers of cytokine responses in a healthy human cohort

**DOI:** 10.1038/s41540-026-00671-w

**Published:** 2026-02-24

**Authors:** Claire Liefferinckx, Jérémie Bottieau, Eric Quertinmont, Vjola Tafciu, Charlotte Minsart, Denis Franchimont

**Affiliations:** 1 Center for the study of IBD, Laboratory of Experimental Gastroenterology, Université libre de Bruxelles, Brussels, Belgium; 2 Department of Gastroenterology, Hepatopancreatology, and Digestive Oncology, HUB Hôpital Erasme, Université Libre de Bruxelles, Brussels, Belgium

**Keywords:** Computational biology and bioinformatics, Diseases, Genetics, Immunology

## Abstract

Population based studies are essential to evaluate the impact of the genetic and environmental determinants that influence the regulation of the human immune response. In a unique and highly selected cohort of healthy subjects, we applied a thoroughly benchmarked machine learning (ML) framework to identify their key predictive drivers following Toll-like receptor (TLR) and T-cell receptor (TCR) stimulations. Patterns of cytokine response, or immunotypes, could be observed across healthy individuals with low and high cytokine producers. Feature importance analysis revealed that TCR-induced predictions were mainly driven by genetic factors, while TLR-induced predictions were predominantly influenced by environmental and biological factors. The best performing model achieved an average correlation of 0.53 for TCR-induced cytokines and 0.27 for TLR-induced responses. Interestingly, adding biological and environmental data to genetic data improved prediction performance by 0.2 on average. However, we showed that ML models using genetic data may overestimate predictive accuracy. These findings were replicated in an independent cohort, the “Milieu Interieur” cohort. Notably, we also showed that polygenic scores for immune-mediated diseases failed to improve model performance, suggesting that the genetics underlying the disease susceptibility do not fully capture the spectrum of functional immune response variability. Our findings define distinct genetic and environmental determinants of cytokine and demonstrate both the values and limitations of ML models for modeling cytokine responses.

## Introduction

The human immune system is a central determinant of health, essential for both host defence and immune tolerance^1^. However, significant inter-individual variability exists in both immune cell composition and responses to pathogens or antigens, making it challenging to clearly distinguish between healthy and pathological immune states^2^. Deciphering the determinants of this immune inter-variability is crucial for understanding the mechanisms underlying immune-mediated diseases (IMIDs) and particularly the wide spectrum of disease severity observed among affected IMID patients. Colocalization studies between genetic variants linked to immune cell traits and genome-wide association studies (GWAS) for IMIDs have enhanced our understanding of their susceptibility and pathogeny^3–7^. However, despite these advances, the variability in IMIDs severity remains poorly captured^8^. One possible explanation is that the inter-individual magnitude of the immune response itself has not been properly assessed in previous studies.

Two large consortia (Milieu interieur cohort and Human Functional Genomics Project) were established to investigate the genetic and environmental factors contributing to the immune response variability^9,10^. Cytokine production following in vitro stimulation has emerged as a key functional phenotype to quantify the magnitude of immune responses. These responses vary greatly between individuals due to a complex interplay of intrinsic and environmental factors^11–13^. Environmental exposures such as cytomegalovirus (CMV) infection^14,15^, smoking^16^, seasonal variation^17^, gut microbiota^18^ have all been shown to significantly modulate cytokine production. Sex-based differences also contribute to variability in immune responses, with women displaying a higher predisposition to autoimmune diseases and men to infections and cancers^19^. From a genetic perspective, several studies have identified common variants associated with cytokine regulation^20–23^. Bakker et al. further advanced this field by integrating multi-omics and deep phenotyping data to build linear predictive models of cytokine responses, laying the foundation for personalized immunology^24^. However, these efforts did not fully leverage machine learning methods for predicting cytokine responses, which could facilitate capturing the interplay between genetic, biological, and environmental factors.

**Fig. 1:**
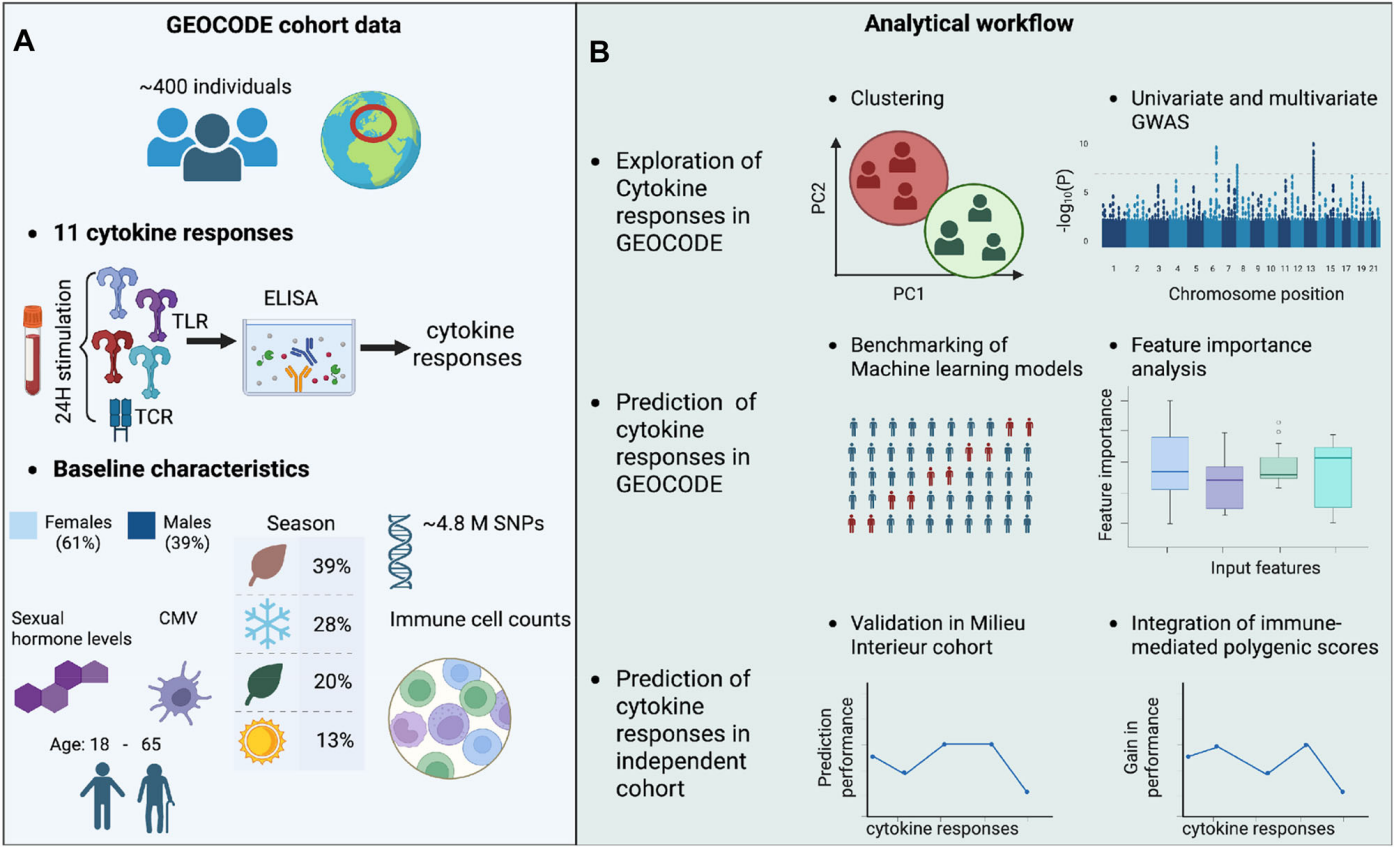
Study data collection and workflow. **A** Data Collection: Data were obtained from 406 healthy individuals in the GEOCODE cohort. Baseline variables included date of sample collection, sex, age, height, weight, immune cell counts, circulating hormone levels, and cytomegalovirus (CMV) serostatus. Genotyping was performed using Illumina Human OmniExpress BeadChips, followed by imputation and quality control. Cytokine responses were measured in whole blood cultures stimulated for 24 h with a panel of Toll-like receptor (TLR) agonists and a T cell receptor (TCR) agonist. **B** Data Workflow: Two complementary analytical approaches were conducted. **(1) Exploratory Analysis:** the goal is to dissect the structure and variability of cytokine responses through the separate application of clustering and both univariate and multivariate genome-wide association studies (GWAS) **(2) Predictive Analysis:** the goal focuses on modeling cytokine production levels based on baseline characteristics (including age, sex, BMI, immune cell counts, seasonal variation, and CMV status) as well as genetic variants. A 5-fold cross-validation scheme was used to evaluate and compare predictive performance across a standard method of clumping and thresholding and machine learning approaches. Feature importance was assessed using the best-performing model, Random Forest, for identifying key predictive drivers of cytokine responses. Created in BioRender. Liefferinckx, C. (2026) https://BioRender.com/1uw7way.

To advance the functional characterization of immune response, we established the GEOCODE cohort, a deeply phenotyped population of healthy individuals on whom we conducted standardized whole-blood stimulation with Toll-like receptor (TLR) agonists and a T-cell receptor (TCR) agonist, leading to the measurement of 11 cytokine responses. Building on this cohort, we expanded the immune cis-regulome using whole-blood transcriptome profiles in ref. ^25^. In this present study (see Fig. 1), we applied a comprehensive analytical framework to dissect the genetic, environmental, and biological determinants of cytokine response variability. We showed two divergent immunotypes for IL6 cytokine responses, while GWAS identified two novel genetic loci associated with cytokine responses, alongside the replication of two known associations. We showed that machine learning (ML) models, particularly random forests (RF), outperformed linear models in predicting cytokine responses when integrating genetic, biological, and environmental factors. Feature importance analysis revealed distinct predictive drivers for TCR- versus TLR-stimulated cytokine responses. Finally, we replicated model performance and feature contributions in the independent Milieu Interieur cohort for four overlapping cytokine traits and evaluated whether polygenic risk scores for immune-mediated diseases could further improve predictive accuracy.

## Results

### Study population

The GEOCODE cohort included 406 healthy individuals, 248 females and 158 males, recruited based on strict inclusion and exclusion criteria previously described^25^. Particular attention was given to the careful selection of participants, excluding known factors that may influence immune responses. A detailed anamnesis was conducted prior to blood collection to avoid recent exogenous immune stimulation (e.g., recent infections or vaccinations). All blood samples were collected in the morning under fasting conditions. The median participant age was 29 years (IQR 25–39), with no significant sex-based differences. However, males had a significantly higher body mass index (BMI) than females (24.4 vs. 23.2; *p* < 0.005). A history of allergy was reported in 4.4% (*n* = 18), although individuals with active allergic conditions were excluded. Among females, 69% (*n* = 171) reported using hormonal contraception. Overall, 3.2% (*n* = 13) of participants were taking permitted medications at the time of sampling (see “Methods”), and none were active smokers. Baseline characteristics are presented as histograms in Supplementary Fig. 1.

Genome-wide genotyping was performed using Illumina Human OmniExpress BeadChips. Following standard single nucleotide polymorphism (SNP) quality control procedures already described^25^, a total of 4,812,146 variants were retained for downstream analyses. The phenotype of the immune response was assessed using stimulated whole blood cultures, considering the type of stimulant, the cytokines measured, and the magnitude of the cytokine response.

Cytokines levels of IL-6, TNF-α, IFN-γ, IL-2, and IL-1RA were measured using Enzyme-Linked Immunosorbent Assay (ELISA) after 24-h incubation of standardized whole-blood cultures using 6 different conditions of stimulations Toll-like receptor (TLR) agonists and T-cell receptor (TCR) agonists (see **Experimental model and study participant details**). In total, 11 cytokine responses were evaluated, with their distributions shown as histograms in Supplementary Fig. 2. The seasonality of sample collection is also shown in Supplementary Fig. 2.

### Clustering analysis uncovers divergent IL-6 Response Phenotypes

**Fig. 2:**
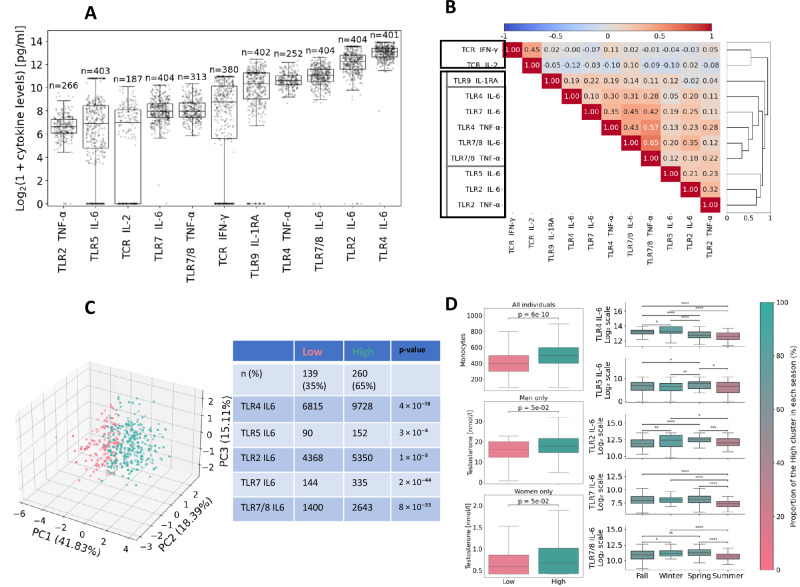
Characterization of cytokine response profiles. **A** Boxplots of 11 cytokine responses measured after 24-h stimulation. The x-axis displays individual cytokines; the y-axis displays log₂-transformed cytokine levels [log₂(1 + cytokine levels)]. The center line shows the median, the hinges represent the first (Q1) and third (Q3) quartiles, and the whiskers extend to 1.5 x IQR or to the minimum/maximum values. The number of non-missing data points is indicated above each boxplot. **B** Heatmap of pairwise Pearson correlations between cytokine responses. Red indicates strong positive correlations; blue indicates strong negative correlations. Hierarchical clustering (right dendrogram) was performed using Pearson correlation as the similarity metric. **C** Hierarchical clustering of 399 individuals based on IL-6 response profiles, visualized using the first three principal components. Clustering was performed using Euclidean distance on IL-6 cytokine responses transformed via inverse rank normalization. Two distinct clusters were identified using both the Gap statistic and silhouette score: the seagreen cluster represents high IL-6 cytokine response profile; the red cluster represents low IL-6 cytokine response profile. The table shows the corresponding median values for both groups together with the p-values from the Mann–Whitney tests. **D** Comparison between high and low IL-6 response groups revealed significant differences in monocyte counts, male sex, and season of sampling. The testosterone analyses shown were performed separately in men and women. Mann–Whitney tests were used. *P* value annotation legend: *: 1e–02 < *p* <= 5e–02, **: 1e–03 < *p* <= 1e–02, ***: 1e–04 < *p* <= 1e–03, ****: *p* <= 1e–04.

A large inter-individual variation in cytokine production was observed across the 11 cytokine responses following immune stimulation (Fig. 2A). A pairwise Pearson correlation analysis, performed on the 11 cytokine responses, revealed distinct co-regulation patterns. Hierarchical clustering on these correlations identified two primary clusters of cytokine responses. These clusters corresponded to T-cell receptor (TCR)-stimulated cytokines (e.g., IFN-γ, IL-2) and Toll-like receptor (TLR)-stimulated cytokines (e.g., IL-6, TNF-α, IL-1RA), as highlighted by the first split in the right dendrogram (Fig. 2B).

Within the TLR-stimulated cytokine cluster, all cytokine responses showed positive correlations, indicating that individuals with a heightened response in one cytokine tended to exhibit elevated levels in the others as well. This observation demonstrates that, for one individual, it seems to exist a specific immune response profile (high or low cytokine producers). Further dissection of the TLR-stimulated responses revealed three distinct patterns (Fig. 2B), each associated with stimulation via different TLRs: (i) IL-1RA response driven by TLR9 activation, (ii) IL-6 and TNF-α responses to TLR4, TLR7, and TLR8, (iii) IL-6 and TNF-α responses to TLR2 and TLR5. To further characterize these patterns of cytokine response to TLR stimulation, which we defined as immunotypes, we conducted an unsupervised clustering analysis of individuals according to their IL-6 cytokine responses **(see Methods section Hierarchical Clustering)**. We focused on IL-6 cytokine responses to define immunotypes because they effectively summarized the main variation across TLR-stimulated cytokines while maximizing sample size. Including additional TCR- or TCR-stimulated cytokines would have reduced sample size without yielding further insight. The number of immunotypes was determined using two evaluation metrics: the average silhouette score and the gap statistic. The average silhouette score reached a maximum at two clusters while the gap statistic shows an increase at two clusters compared to the one-cluster solution (Supplementary Fig. 3). These two clusters are visualized in Fig. 2C, where individuals are projected onto the first three principal components of the IL-6 cytokine responses. The cluster in red, representing 35% of participants (*n* = 139), exhibited a lower IL-6 producer phenotype, while the other in green displayed elevated IL-6 production phenotype (*n* = 260). Factors significantly associated with the high IL-6 cluster included elevated monocyte counts (*p* < 0.001), male sex (*p* < 0.001), and sample collection during the winter season (*p* < 0.001). Regarding the influence of hormones, although testosterone levels differed significantly between sexes, higher testosterone levels were associated with increased IL-6 production in both males and females (Fig. 2D and Supplementary Table 1). Interestingly, no association with oestradiol levels was found, although ref. ^26^ suggested that the higher IL-6 production observed in males may be partly explained by the suppressive effect of estrogen receptors on IL-6 expression. Seasonal variation in IL-6 levels has also been described^27^. Moreover, the relationship between IL-6 production and monocytes is particularly notable, as monocytes are a source of IL-6. However, IL-6 also has the capacity to modulate immune responses by influencing the differentiation of immune cells, such as promoting the transition from neutrophils to monocytes, making this association more complex and bidirectional^28^.

### Cytokine response loci uncovered through univariate and multivariate genome-wide association studies

**Fig. 3:**
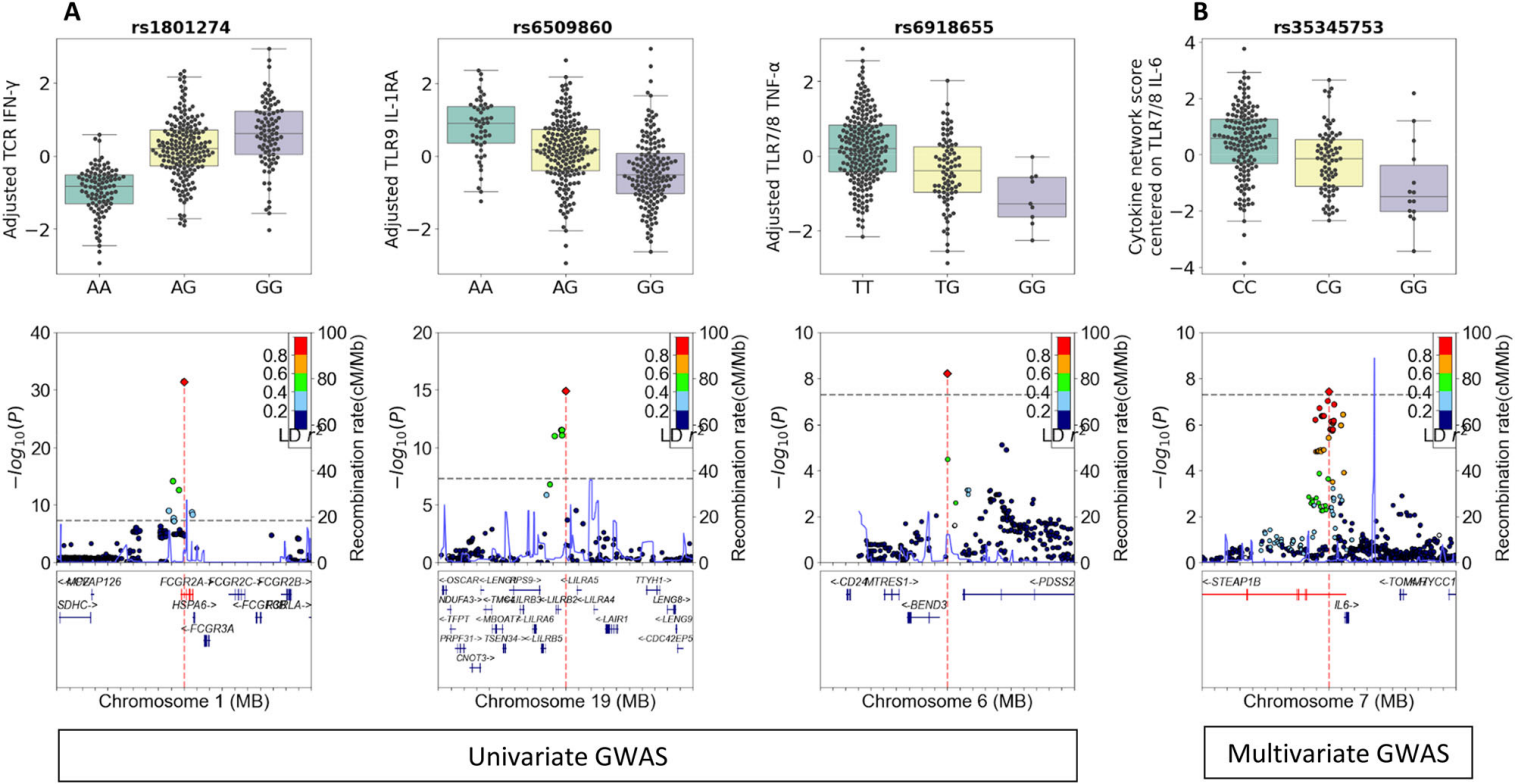
Genome-wide significant associations (GWAS) between genetic variants and cytokine responses. **A** Univariate GWAS. Box plots and locuszoom plots showing the association of 4 genome-wide significant (*p* value < 5.10^( − 8)) Protein Quantitative trait loci (pQTL): chromosome 1 SNP chr1:161509955 with TCR-IFN, chromosome 19 SNP chr19:54288504 with TLR9-IL1Ra, chromosome 6 SNP chr6:107127476 with TLR7/8-TNF, The box length represents the interquartile range (IQR, Q3–Q1), while the whiskers extend up to 1.5 × IQR from the edges of the box. **B** Multivariate GWAS. Box plot and locuszoom plot showing the association of one genome-wide significant (*p* value < 5.10^( − 8)) Protein Quantitative trait loci (pQTL): chromosome 7 SNP rs74513903 with the cytokine network score centered on the TLR 7/8 IL-6. The boxplots show adjusted cytokine production stratified by the genotype of the top SNP identified in the GWAS. The cytokine production has been adjusted for age, sex, BMI, peripheral immune cell counts, and season. The color scale indicates the level of linkage disequilibrium (LD) between SNPs. The lower panel of the LocusZoom plot displays the genomic distribution of genes, with genes shown in red when the SNP is located within the gene.

We tested 4,812,146 high-quality imputed genetic variants for association with cytokine responses under each condition of stimulation, adjusting for age, sex, BMI, peripheral immune cell counts, and season. A genome-wide significance threshold of p < 5 × 10⁻⁸ was applied. Both univariate and multivariate genome-wide association analyses were performed (see **Methods section GWAS analysis**), as multivariate approaches have been shown to enhance statistical power, particularly in small sized cohorts. Given missing data for several cytokine responses, we performed GWAS on both non-imputed and imputed datasets. The imputed dataset was used to maximize sample size, following the phenotype imputation strategy proposed by Dahl et al.^29^. Importantly, this imputation was used exclusively for the GWAS analyses and not for the ML prediction analyses (see **Methods section Cytokine response imputation** and Supplementary Fig. 4). We identified a total of four cytokine response-associated loci reaching genome-wide significance that were common to both the imputed and non-imputed datasets (Fig. 3). Quantile-quantile (QQ) plots evaluating the genomic inflation are shown in Supplementary Fig. 5. Interestingly, three additional loci marginally surpassed genome-wide significance in the non-imputed GWAS analyses, but were not retained, as these signals were not replicated in the imputed analyses.

In the univariate GWAS, three genome-wide significant loci were identified: rs1801274 (chr1:161509955), associated with IFN-γ and IL-2 responses to TCR stimulation; rs6509860 (chr19:54288504), associated with IL-1RA response to TLR9 stimulation; and rs6918655 (chr6:107127476), associated with TNF-α response to TLR7/8 stimulation. The association of rs1801274 with IFN-γ and IL-2 responses to TCR stimulation is well established and has been previously reported and confirmed^25,30,31^. The association between rs6509860 and IL1-RA as well as rs6918655 and TNF-α have not yet been described.

To exploit the correlation between cytokine responses, we employed a multivariate GWAS framework using canonical correlation analysis, as implemented in MV-PLINK^32^. For each genetic marker, its association with a linear combination of a set of cytokine responses, referred to as a cytokine network, was tested, which maximizes the correlation with the given marker. We defined 11 different cytokine networks, each centered on a specific cytokine response. Each cytokine network was constructed by including cytokine responses whose absolute Pearson correlation with the central cytokine response exceeded 0.2 (see Supplementary Table 2).

Using this multivariate GWAS approach, we identified one additional genome-wide significant locus associated with the cytokine network centered on the IL-6 response to TLR 7/8, while the other genome-wide cytokine response loci from the univariate GWAS were either significant or suggestive in the multivariate analyses (see Supplementary Fig. 6). MV-PLINK provides loadings for each of the 5 cytokines within the cytokine network, indicating their respective weight to the linearly combined trait tested for association (see Supplementary Table 3). IL-6 responses to TLR7 (gardiquimod) and TLR7/8 (resiquimod) showed the highest loadings (0.72 and 0.62, respectively). The lead SNP, rs35345753 (chr7:22700894) at this locus lies in close vicinity to the IL-6 gene, further supporting its biological relevance in modulating TLR-induced cytokine responses (Fig. 3). Notably, this variant has also been associated with IL-6 production in the Milieu Intérieur study, further validating the robustness and relevance of this genetic association^16^.

### Machine-learning benchmark of cytokine response prediction

Seven predictive models were benchmarked on the 11 cytokine responses in the GEOCODE cohort with a five-fold cross-validation strategy. One used the clumping and thresholding (C + T) method, while the other six, including ordinary least squares, ridge and elastic net regression, random forest (RF), gradient boosted trees (GBT), and artificial neural networks (ANN), were based on supervised machine learning (ML) approaches. In a first step, the models were compared using only genetic markers as input variables, before being compared again using both genetic and biological/environmental data (see **Methods section Predictive modeling framework within GEOCODE**). For result visualization, we retained the Elastic Net model among the linear models, given that all linear models yielded comparable performance across all cytokine responses (see Supplementary Fig. 7).

**Fig. 4:**
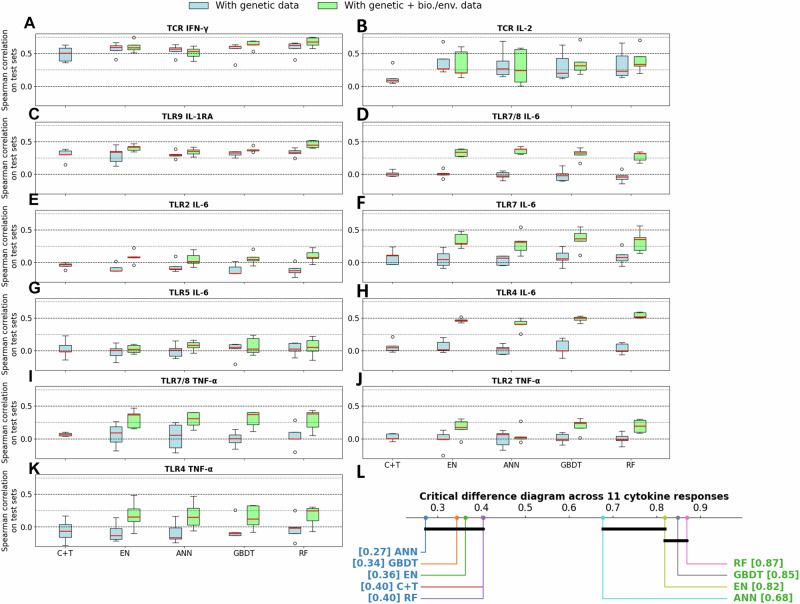
Machine-learning benchmark across 11 cytokine responses. **A**–**K** Comparison of test performance across five-fold cross-validation for the five predictive models using either genetic data only (blue) or combined genetic, biological, and environmental data (green), evaluated for each of the 11 cytokine responses. Test performance is reported as Spearman correlations on the test sets and visualized as boxplots. EN denotes the linear Elastic Net model, ANN the artificial neural network, GBDT the gradient boosted trees, and RF the random forest. **L** Critical difference diagram summarizing the average ranks of all models across the 11 cytokine responses. Significant pairwise differences were evaluated using Conover post-hoc comparisons following Friedmans’ test (*p* = 2.3 × 10–9). Models that cannot be statistically distinguished at α = 0.05 are connected by horizontal bars.

Figure 4A–K compares the predictive performance, measured by Spearman correlation, of the five ML models across the 11 cytokine responses, using either genetic markers only or combined genetic, biological, and environmental data as input features. Figure 4L shows the corresponding critical difference diagram, which summarizes the pairwise statistical comparison among the 9 versions of the predictive models across the 11 cytokine responses.

Focusing on models trained with genetic data only (shown in blue in Fig. 4A–K), the C + T method showed its highest average correlation for the TCR-activated cytokines IFN-$\gamma$ (0.49) and IL-2 (0.13), and for IL-1RA stimulated by TLR9 (0.30), suggesting that predictive potential of genetic markers was limited to these three cytokine responses. For TCR activations, the Elastic Net (EN) linear model, which accounts for joint effects of genetic markers, shows average improvements over the C + T method, with gains of 0.23 for IL-2 and 0.07 for IFN-$\gamma$. The inclusion of non-linear models (RF, GBT, and ANN) leads to similar performance to linear models, without providing substantial gains when using only genetic markers. Overall, this is corroborated in Fig. 4L, which shows that the models trained with genetical-only data cannot be statistically distinguished from one another, with C + T and RF ranked the highest. Looking at the effect of adding biological and environmental as input features (shown in green in Fig. 4A–K), the EN linear model shows clear improvement over its genetic-only counterpart, particularly for TLR-stimulated cytokines, with an average improvement of 0.23 across all TLR responses. The highest gain was observed for IL-6 stimulated by TLR4 ( + 0.40), while the lowest was for IL-6 stimulated by TLR5 ( + 0.04). Using the EN linear model with combined input features as the baseline, non-linear models show distinct behavior in their average improvement. RF and GBT demonstrated more consistent performance gains across cytokine responses (average improvements of +0.02 for RF and +0.01 for GBT), while the ANN exhibited an average decrease of -0.04. This greater consistency of RF and GBT is further supported by Fig. 4L, where these models achieve the highest average rank across the 11 cytokine responses, with RF ranking first overall. RF is statistically better than ANN, but not significantly different from EN and GBDT. For visualization, the predictions of the RF model with the actual measured cytokine responses are plotted across the 5 data splits used for cross-validation in Supplementary Fig. 8.

### Key predictive drivers of TCR- and TLR- stimulated cytokines are different

**Fig. 5:**
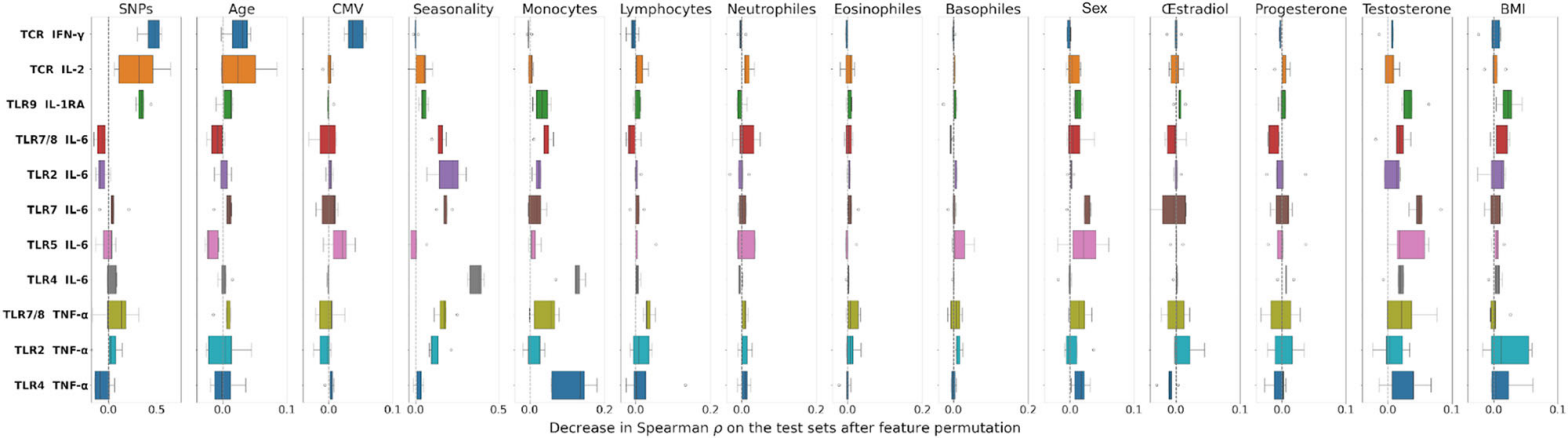
Variable importance in predicting cytokine responses using random forest. Relative importance of input variable categories for predicting 11 cytokine responses, assessed using Random Forest models on test sets from cross-validation on the GEOCODE cohort. Importance is estimated based on the decrease in predictive model performance (Spearman correlation) observed when a given variable is randomly permuted, thus reflecting its contribution to the prediction.

As the RF model proved to be the most reliable, we further investigated the relative importance of its input variables (Fig. 5). This importance was estimated statistically by measuring the decrease in model performance, measured via Spearman’s correlation on data used for evaluation (the five test sets), when the values of a given input variable were randomly permuted (see Feature importance analysis in Methods). An importance score near 0 indicates that the input feature has little impact on model performance, while an importance score close to 1 denotes a major influence on the model’s performance. For clarity, the effects of genetic markers were summed into a single category referred to as ‘SNPs’. As with the overall predictive performance, a clear dichotomy was observed between cytokine responses triggered by TCRs and those induced by TLRs.

For TCR-driven responses, SNPs emerged as the most predictive features, with a moderate effect of age. CMV serostatus also had a modest influence for IFN-$\gamma$ prediction. In contrast, TLR-induced cytokines were more strongly influenced by environmental and biological variables, especially seasonality and monocyte counts. Notably, importance for monocytes peaked for TLR4-stimulated cytokines. Interestingly, the TLR9 IL-1RA response showed a mixed profile, where both SNPs, and to a lesser extent, seasonality and monocytes contribute to the prediction. Finally, TLR5 IL-6 was poorly predicted by any of the input features, suggesting limited explanatory power with the available input variables. In addition, sex and sexual hormones, particularly testosterone, exerted a modest influence across most cytokine responses. Finally, BMI appeared to play a minor role, primarily for IL-1RA activated by TLR9 and TNF-α activated by TLR2 and TLR4. This limited contribution likely reflects the GEOCODE cohort’s relative BMI homogeneity, stemming from its inclusion criteria.

Finally, we evaluated to what extent the importance of SNPs can be artificially inflated in predictive models due to data leakage during genetic feature selection (see Supplementary Fig. 9). Data leakage occurs when information from the test set inadvertently influences the model training process, leading to overoptimistic performance. Specifically, we compared the performance of the RF model under two scenarios (see **Data preprocessing in Methods**): (i) our reference scenario in which the genetic feature selection was restricted to the training set only, and (ii) a data-leaking scenario where genetic markers were selected using the entire cohort (including the test set). As shown in **the upper panel of** Supplementary Fig. 9, the data-leaking scenario leads to a noticeable inflation in model performance, yielding systematically higher Spearman correlations, with an average increase of 0.25 (green boxplots). **The lower panel of** Supplementary Fig. 9 illustrates that this performance gain is primarily driven by a greater importance of SNPs for the data-leaking scenario, with their importance rising by an average of 0.15. This highlights how easily the importance of genetic markers can be overestimated in predictive frameworks when proper precautions, such as maintaining a strict separation between training and test data during genetic feature selection, are not observed.

### External validation of the predictive models on the Milieu Interieur cohort

To externally validate our predictive models, we applied the best-performing model, the RF model, to an independent cohort: the Milieu Interieur cohort. Only non-smokers were retained from the Milieu Interieur Cohort (n = 497). Prior to prediction, we assessed the comparability of the two cohorts (GEOCODE and Milieu Interieur cohorts) using shared phenotypic and genetic data (Supplementary Fig. 10). Notably, four cytokine responses overlapped between the two datasets, involving the responses of IL-6, IL-2, IFN-γ, and TNF-α. Notably, the two cohorts differ for TLR4 (LPS) stimulation of IL-6 and TNF-α due to varying LPS concentrations used for stimulation (3 μg/ml in the Geocode cohort and 10 μg/ml in the MI cohort). As a result, a prediction model trained on the GEOCODE data cannot replicate the real-value distribution seen in the MI cohort. However, since the evaluation metrics are rank-based, this difference in absolute values does not affect the results.

**Fig. 6:**
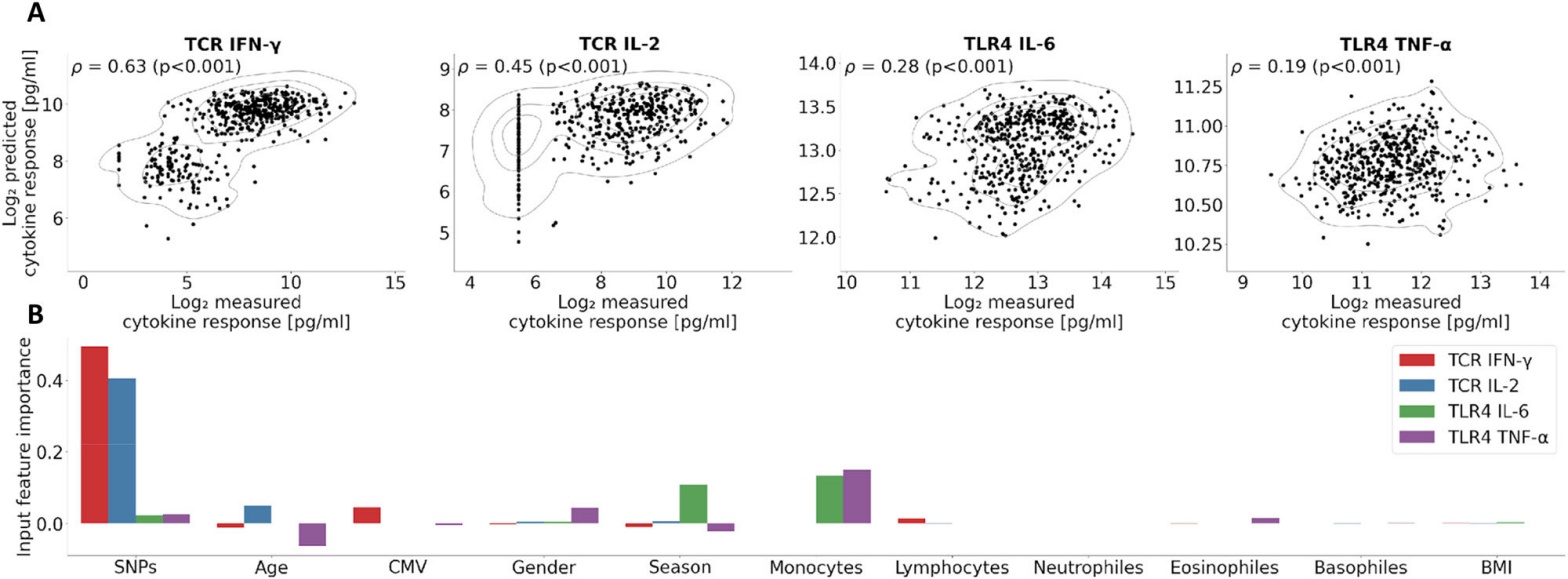
Cross-cohort prediction of cytokine responses and variable importance. **A** Predictive performance of the Random Forest (RF) model trained on the GEOCODE cohort and tested on the Milieu Intérieur (MI) cohort for four shared cytokine responses. Each subplot shows the observed vs predicted log₂-transformed cytokine levels in the MI cohort, with density contour lines. Spearman’s correlation coefficient (ρ) and associated *p* value quantify the monotonic relationship between predictions and actual measurements. **B** Relative importance of different categories of input variables used in the RF model for each cytokine response, estimated by the decrease in Spearman correlation observed after randomly permuting each variable.

The RF model was trained on the full GEOCODE cohort and then used to predict cytokine responses in the Milieu Interieur cohort (see **Methods section Predictive modeling framework for MI cohort**). The list of selected genetic markers is provided in Supplementary Table 4. Predictive performance was successfully replicated, with Spearman correlation coefficients ranging from 0.19 to 0.63 (Fig. 6A). Importantly, similar trends in model performance were observed in this independent cohort: predictions for TCR-induced cytokines (IL-2 and IFN-γ) were consistently more accurate than those for TLR-induced cytokines (IL-6 and TNF-α). While such models can rank individuals based on their predicted cytokine responses, they output cytokine concentrations within the range of their training data and therefore cannot extrapolate the concentration gap between GEOCODE and MI for TLR4 stimulations

Moreover, the feature importance profiles mirrored those observed in the GEOCODE cohort (Fig. 6B). Specifically, TCR-stimulated cytokine responses (e.g., IL-2 and IFN-γ) were mainly driven by genetic factors, age, and CMV serostatus, whereas TLR-induced cytokine responses were more strongly influenced by seasonality and monocyte counts.

### Immune mediated disease risk loci show limited predictive value for cytokine response

To investigate the potential mechanistic link between genetic susceptibility to immune-mediated inflammatory diseases (IMIDs) and cytokine responses in healthy individuals, we analyzed polygenic scores (PGS) for several IMIDs: inflammatory bowel disease (IBD), with ulcerative colitis (UC) and Crohn’s disease (CD), multiple sclerosis (MS), psoriasis, type 1 diabetes (T1D), and rheumatoid arthritis (RA) (Supplementary Table 5). These scores were derived from the *Polygenic Score Catalog*^33^ and applied to both the GEOCODE and Milieu Interieur cohorts (see **Methods section PGS calculation**).

**Fig. 7:**
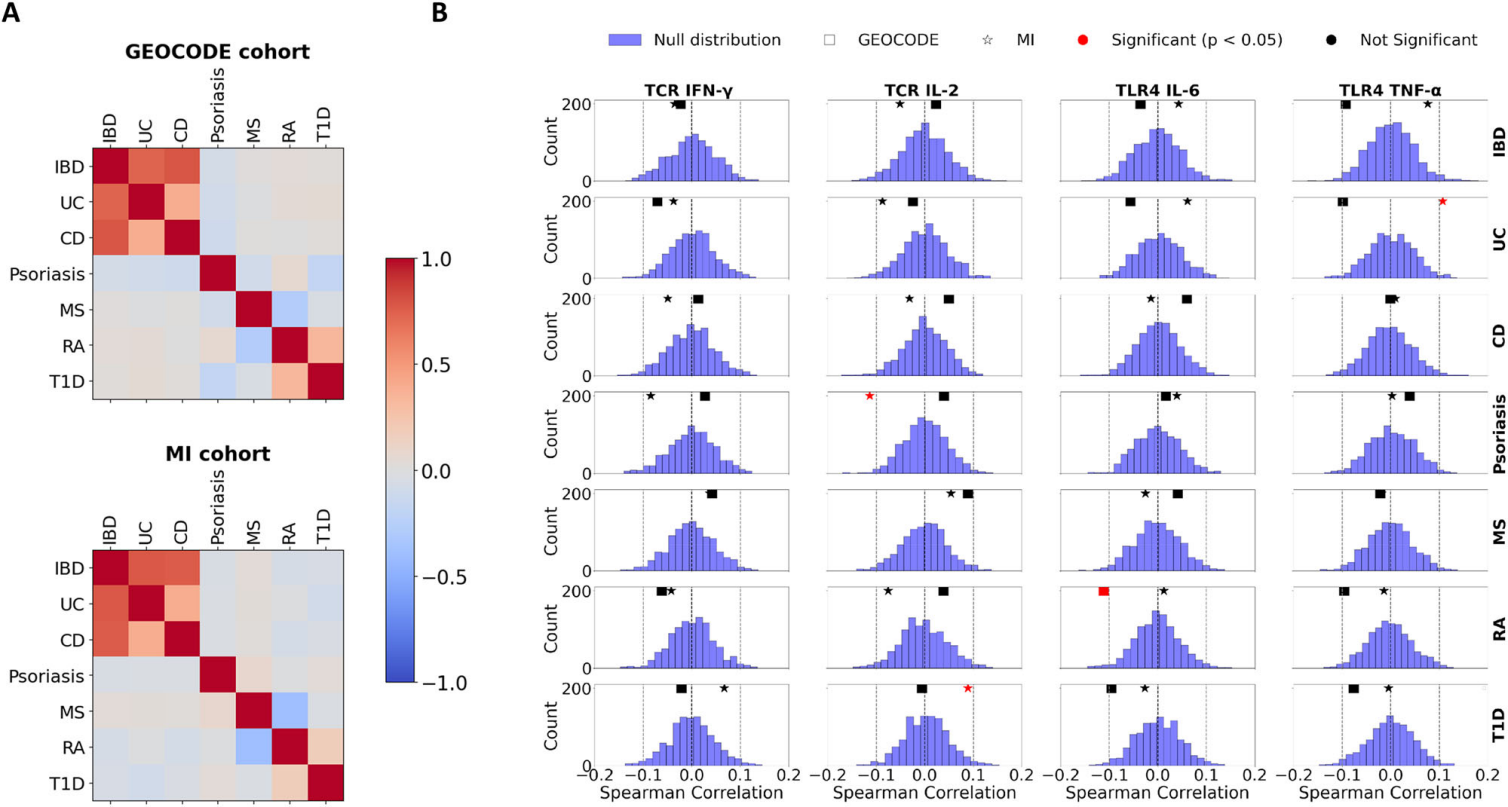
Associations between immune-mediated inflammatory diseases (IMID) polygenic scores and cytokine responses. **A** Heatmaps showing correlations between polygenic scores (PGS) for IMIDs (Inflammatory bowel disease, Ulcerative colitis, Crohn’s disease, psoriasis, multiple sclerosis, rheumatoid arthritis, and type 1 diabetes) in the GEOCODE and Milieu Intérieur (MI) cohorts. PGSs were derived from the *Polygenic Score Catalog*. **B** Spearman correlations between cytokine responses and IMIDs PGSs in both cohorts. Squares (GEOCODE) and stars (MI) represent observed correlations. Null distributions (based on 1000 permutations) are shown for MI. Red markers denote statistically significant correlations (*p* < 0.05), while black markers indicate non-significance.

As shown in Fig. 7A, the correlation structures between IMIDs PGS were largely consistent across both cohorts. We next evaluated the associations between IMIDs PGS and cytokine responses in both cohorts (Fig. 7B). Measured correlations were compared against a null distribution obtained by 1000 random permutations (see **Methods section Association between PGS and cytokine responses**). None of the nominally significant associations were consistently replicated between cohorts. This suggests that, using this approach, it is difficult to identify consistent overlap between genetic risk for IMIDs and cytokine response variability in healthy individuals.

To further assess the predictive utility of PGS, we integrated them into our RF models as additional input variables (Supplementary Fig. 11). The models, trained on the GEOCODE cohort and tested on the Milieu Interieur cohort across four shared cytokine responses, showed no meaningful improvement in predictive performance when IMIDs PGS were included. Feature importance analysis confirmed that IMIDs PGS had minimal influence, with near-zero contribution across cytokine traits. These findings underscore the complexity of immune regulation and suggest that, in the context of healthy individuals, common IMID risk variants may not capture the functional variability in cytokine production.

## Discussion

Cytokine responses are shaped by a complex interplay of biological, environmental, and genetic factors. In our study, we observed that cytokine responses triggered by TLR stimulation were predominantly influenced by environmental and biological factors such as seasonality and monocyte counts whereas TCR-induced responses were more strongly associated with genetic variation. Our results mirror findings from twin studies where IL-2 showed high heritability, whereas IL-6 was largely non-heritable^11^. Seasonality emerged as a major determinant of IL-6 production and significantly contributed to predictive models, consistent with previous studies^13,17^. In contrast, age had a weaker effect, likely due to the age homogeneity of our cohort^12,13^. Although we identified a genome-wide significant locus associated with IL-6 response, the aggregated genetic contribution to IL-6 response appeared negligible in terms of predictive performance. This aligns with the ongoing debate about IL-6 heritability, which may be context-dependent and stimulus-specific^11,21,24^. Linear mixed models, often used to estimate heritability, face limitations in modestly sized cohorts (< 1.000 samples), producing wide confidence intervals that hinder precise estimation^34^. Even for a highly specific immune phenotype such as IL-6 response under controlled in vitro conditions, heritability remains difficult to estimate. This finding challenges the intuitive assumption that such a well-defined trait would be governed by a few strong-effect loci. One can hypothesize that IL-6 response is either only modestly heritable, or follows a polygenic model, in which numerous small-effect variants, in addition to core immune genes, contribute to its regulation. In contrast, IL-2 and IFN-γ responses appear to be predominantly governed by a limited number of moderate-effect loci. We confirmed the influence of a key genetic variant with pleiotropic effects, rs1801274, which was found to influence both cytokine production and lymphocyte subset distribution^6^. Additionally, cytomegalovirus (CMV) serostatus contributed to inter-individual variation in TCR-induced cytokine responses, in line with previous reports^11^. Although our study was not designed to dissect mechanisms underlying the observed associations, our findings are consistent with several previously studies. Persistent viral exposures such as CMV infection have been shown to strongly shape T cell composition and responsiveness, providing a plausible explanation for the variability observed in TCR-stimulated cytokine responses^35^. In parallel, variation in TLR-driven cytokine responses may reflect cumulative environmental and microbial exposures acting on the innate immune system, a process related to trained immunity, whereby prior stimulation leads to long-lasting functional reprogramming of innate immune cells^36^. More generally, recent theoretical and experimental work suggests that stable immunotypes can emerge from the dynamic interplay between genetic background, exposome, and immune regulation, and can be maintained over time through homeostatic mechanisms^37,38^. While our data are compatible with these observations, longitudinal and mechanistic studies will be required to directly confirm these hypotheses.

Developing predictive models of cytokine responses may help extrapolate findings from immune heterogeneity observed under controlled conditions to pathological contexts such as in IMIDs. However, such extrapolation is only meaningful if model performance is sufficient, which requires benchmarking. In brief, we compared predictive models of increasing complexity (ranging from the clumping and thresholding (C + T) method to linear models and ML methods) across 11 cytokine responses. Models were evaluated on two types of input data: genetic-only and genetic with biological/environmental data. For scalability and comparability concerns, all models were evaluated using the C + T-selected genetic markers. The best-performing model, RF, achieved results that were successfully replicated in the Milieu Interieur cohort. On genetic-only data, only 3 out of 11 cytokines achieved a positive average Spearman correlation across predictive models. For TCR activations, both linear and ML models outperformed the C + T baseline, which is consistent with previous results, such as those by ref. ^39^ on blood cell traits, where learning-based approaches outperformed C + T. The improved performance reflects their ability to account for linkage disequilibrium structure, genetic interactions, and rare variants. Explicitly modeling non-linear effects via ML models did not lead to improved performance, aligning with benchmark studies, which report that ML models (particularly deep learning) do not necessarily outperform linear models by a sizable margin when using genetic-only data^40,41^. However, integrating biological and environmental features with genetic data improved model performance, particularly for TLR-stimulated cytokines. In this setting, tree ensemble models emerged as reliable predictors across 11 cytokine responses. Although genotype-by-environment prediction has not been extensively studied for human traits, these findings are in line with results for crop traits, where tree ensemble models have demonstrated strong predictive performance when integrating genetic and environmental data^42^. In our benchmark, neural networks showed inconsistent predictive performance across the cytokine traits. In such tabular data settings, tree ensemble models often outperform deep neural networks, as demonstrated in broader ML benchmarks^43^. This inconsistency of neural networks likely reflects the challenges in configuring their architectures, which often require extensive hyperparameter tuning. We used random grid search for model hyperparametrization, which is known to offer a good balance between computational efficiency and performance^40^. One promising direction for improving the performance of neural networks is the incorporation of biological priors into the architecture for limiting the number of trainable parameters^41,44^.

Phenotype prediction using genetic data is a high-dimensional problem, where the number of genetic variants far exceeds the number of samples. Feature selection is therefore a crucial step and, in our case, was performed using the C + T method. However, this type of filtering may introduce data leakage, especially when selecting the genetic markers using the complete dataset, as highlighted by ref. ^45^. We illustrated this issue within our own cohort, where RF models trained with data leakage exhibited consistently better predictive performance across all cytokine responses, and a much stronger apparent contribution from genetic markers. This type of overfitting can occur easily and was likely a factor in the study by ref. ^24^, where strong in-sample predictive performance failed to replicate in an independent dataset.

Shared genetic architecture between immune traits (such as basal plasma protein or blood cell traits) and IMID susceptibility has been well documented^3,46^ but direct links between IMID-associated genetic risk and cytokine production remain less explored due to the complexity of implementing functional immune profiling frameworks^21,25,30^. Bakker et al. reported associations between polygenic scores (PGS) for several IMIDs and stimulated cytokine levels, suggesting shared genetic signals^24^. Their findings suggested disease-specific patterns: some IMIDs, including inflammatory bowel disease, multiple sclerosis and psoriasis showed stronger correlations with lymphocyte-derived cytokines, while others, such as type 1 diabetes and rheumatoid arthritis, were more associated with monocyte-derived cytokines. In contrast, we did not replicate these associations in either our own cohort or the Milieu Intérieur cohort. Several factors may explain this discrepancy. First, our PGS were derived from public datasets, whereas Bakker et al. generated their own, potentially yielding distinct representations of IMID genetic architecture. Second, while Bakker et al. averaged correlations across lymphocyte- and monocyte-derived cytokine groups, our analysis was performed at the level of individual cytokine responses, potentially missing weaker associations at the cell-type levels. However, by including replication across two independent cohorts, we observed that some correlations in one cohort were reversed in the other, an observation that would have been unnoticed without replication. Third, emerging evidence suggests that genetic factors driving IMID susceptibility differ from those influencing disease severity^8,47,48^. Therefore, the limited overlap between cytokine-response genetics and IMID susceptibility may be not unexpected. Rather, our findings underscore the importance of dissecting the genetic and environmental drivers of cytokine production as a foundational step toward understanding the determinants of IMID severity.

In conclusion, we provided a comprehensive analysis of ML methods for predicting cytokine responses, highlighting both their advantages and limitations. We demonstrated the added value of ML models when integrating genetic, biological, and environmental data to predict cytokine responses, particularly for TLR-induced responses, where the genetic signal is harder to pinpoint accurately. We also showed that for genetic data alone, linear models may already extract most of the predictive signal. However, if genetic marker selection and model testing is not properly separated, ML approaches can yield overly optimistic estimates of genetic contributions. Overall, tree-based ensemble methods, specifically RF, outperformed neural networks in this tabular data setting. This work advances our understanding of how well cytokine responses can be predicted and underscores the importance of accounting for both host factors and seasonality when modeling cytokine responses.

## Methods

### Ethics statement

The study cohort (GEOCODE cohort) was approved by the Ethics committee of Erasme Hospital, Brussels, Belgium (Reference number: P2015/425, date approval: 03/11/2015). All used methods were in accordance with approved guidelines and were performed in accordance with the Declaration of Helsinki. Each subject signed an informed consent.

### Study cohorts

The study cohort (GEOCODE cohort) involved 406 healthy subjects prospectively included between October 2016 and March 2018. The full details on the criteria of GEOCODE building have been published elsewhere^25^.

The Milieu Intérieur cohort was developed by the Institut Pasteur team in Paris, France, and consists of a reference population of 1000 healthy donors. This cohort offers a framework for investigating the determinants of human immunological variability, as previously described in detail^9^. Phenotypic, biological, and genetic data were accessed under a data transfer agreement and used to externally validate the predictive models developed in the presented work.

### Sample collection, whole blood stimulation and cytokine profiling

For all participating subjects, 40 ml of peripheral blood were collected between 07h30 and 10h00 a.m. (to standardize the circadian cycle) after overnight fasting. A fresh EDTA tube was processed within the same day of blood collection for immunophenotyping. Details on immunophenotyping have been published elsewhere^49^.

Whole blood cell cultures and simulations were performed within three hours of blood collection. Six different TLR agonists, Resiquimod (R848), 200 ng/ml (InvivoGen)—TLR7/8; Lipopolysaccharide (LPS) from *E. coli* O111:B4 (LPS-EB ultrapure), 3 ng/ml (InvivoGen)—TLR4; Gardiquimod (GAR), 1 µg/ml (InvivoGen)—TLR7; Flagellin (FLA) from *Salmonella typhimurium* (Fla-ST ultrapure), 100 ng/ml (InvivoGen)—TLR5; Lipoteichoic acid (LTA) from *Bacillus subtilis* (LTA-BS), 1 µg/ml (InvivoGen)—TLR2; CpG ODN (ODN) type A (ODN2216), 1 µg/ml (Invivogen) - TLR9; and one TCR antagonist (anti-CD3 (clone UCHT1)/anti-CD28), 3 µg/ml + 1 µg/ml (BioLegend) were used. The IC50 dose (or concentration) of each stimulant was chosen from a ranging dose-stimulation pilot study. Briefly, the blood was diluted 1/4 with pre-warmed FBS-RPMI. TLR agonists or TCR antagonists were added prior to incubation for 4 h and 24 h, at 37 °C in 5% CO2 containing atmosphere. Whole blood from each well was next transferred into a pre-labeled 1.5 ml Eppendorf tubes. After centrifugation, the supernatants were collected and stored at –80 °C until use.

Cytokine production following TCR and TLR stimulations were measured using a standard ELISA method. IL-6 (DuoSet Human IL-6, R&D Systems), TNF-〈 (DuoSet Human TNF-〈, R&D Systems), and IL1-RA (DuoSet Human IL1-RA, R&D Systems) were measured for TLR stimulation conditions. IFN-© (DuoSet Human IFN-©, R&D Systems) and IL-2 (DuoSet Human IL-2, R&D Systems) were measured for TCR stimulation conditions. All cytokine measurements were expressed in pg/ml.

### DNA extraction

Human genomic DNA was extracted from EDTA-collected peripheral blood by automated genomic DNA isolation Tecan Freedom. The extraction was performed in batches of 32 samples. Subsequently, concentration and quality DNA were measured by nanodrop ND-1000. DNA concentration standardized for all samples to 50 ng/ml.

### SNP genotyping and imputation

The 406 individuals were genotyped for >700 K SNPs using Illumina’s Human OmniExpress BeadChips, an iScan system and the Genome Studio software (GIGA genomics core facility, Liège, Belgium). We eliminated variants with call rate ≤0.95, with the minor allele frequency (MAF) < 0.05 and deviating from Hardy–Weinberg equilibrium (HWE) (<0.001). European ancestry of all individuals was analyzed by PCA using the HapMap population as reference and used the 3 first PC as covariates. We used The Michigan Imputation Server with TOPMed Imputation Reference panel (https://imputation.biodatacatalyst.nhlbi.nih.gov) to impute genotypes at autosomal variants in our population. We removed all SNP with info < 0.4. A new quality control has been applied after imputation by filtering all SNP with MAF < 0.05 and HWE < 0.001. Finally, 4,812,146 variants were obtained after imputation.

### Seasonality modeling

Seasonality was modeled following the approach described in ref. ^13^. Two periodic functions were used, based on the day of the year (doy) corresponding to each sample’s collection date: ${s}_{1}=\sin (\frac{2* \pi * {doy}}{365})$ and ${s}_{2}=\cos (\frac{2* \pi * {doy}}{365})$. These two functions represent each sample on the unit circle, where one full revolution corresponds to a one-year cycle (Supplementary Fig. 2).

### Hierarchical clustering

The *AgglomerativeClustering* algorithm from the scikit-learn Python package was used to group individuals based on their IL-6 cytokine response profiles. Prior to clustering, the IL-6 cytokine responses were inverse rank normalized and forced to follow a Gaussian distribution. To select the number of clusters, two evaluation metrics were computed: the average silhouette and the gap statistic. The number of clusters varied from 2 to 15. The silhouette score was computed using the *silhouette_score* function from the scikit-learn Python package. The gap statistic was estimated following the approach described in ref. ^50^, using 100 Monte Carlo reference datasets. In each reference dataset, IL-6 cytokine responses were sampled independently from a uniform distribution spanning the observed range of each feature. The same normalization procedure applied to the original data was also applied to the reference datasets. The silhouette score dropped after two clusters, while the gap function showed an increase when moving from one to two clusters (Supplemental Fig. 3). To compare the two identified clusters, chi-square tests were used for categorical variables and Mann–Whitney U tests for continuous variables (Supplemental Table 1). The p-values were adjusted for multiple testing using the Bonferroni correction. For visualization, Principal Component Analysis (PCA) was performed using the *PCA* class from the scikit-learn Python package.

Note that the *AgglomerativeClustering* algorithm from the scikit-learn Python package was also used to re-order the matrix of Spearman correlations between cytokine responses, where the average linkage method was applied to determine how clusters are merged.

### Cytokine response imputation

From the initial cohort of 406 individuals, we excluded those with more than 80% missing cytokine responses. For the remaining 403 individuals, missing data were imputed using *SoftImpute* algorithm, following the approach proposed by Dahl et al.^29^. The algorithm, implemented via the Python library *fancyimpute*, was applied to the eleven cytokine responses along with available baseline characteristics, including age, BMI, sex, seasonality, immune cell counts, hormones, and CMV serostatus. Prior to imputation, all variables were standardized using the *StandardScaler* from the Python Library scikit-learn. The regularization parameter for the *SoftImpute* algorithm was tuned using this formula: $\lambda ={\Vert {P}_{\varOmega }\left(X\right)\Vert }_{2}/K$, where K takes values from the set [1.5, 2, 4, 10, 50, 100], and ${\Vert {P}_{\varOmega }\left(X\right)\Vert }_{2}$ is the largest singular value of the input matrix *X* (with missing values padded with zeros).

To evaluate imputation accuracy, we randomly masked one observed value per column and calculated the mean squared error (MSE) between the true and imputed values. This procedure was repeated 1000 times for each $\lambda$ value, and the average MSE was used to determine the optimal regularization parameter, which was found to be $\lambda =3.63$. Supplementary Fig. 4A shows the averaged MSE obtained for the different values of $\lambda$. Supplementary Fig. 4B highlights the relative improvement in imputation accuracy, expressed as a percentage, compared to mean imputation across all cytokine responses, using the root mean squared error (RMSE) skill score, for the optimal $\lambda$ value.

Imputation was applied to cytokine traits prior to GWAS to maximize sample. This approach yields more conservative association signals in our cohort. Imputation of missing cytokine values was performed exclusively for the GWAS analyses, whereas all machine-learning models were trained and evaluated using only complete-case datasets without any imputation.

### GWAS analyses

Before performing association analyses, the effects of sex, age, BMI, seasonal variation, immune cell counts, and the first three principal components reflecting genetic ancestry were regressed out from each imputed cytokine response. The resulting residuals were then transformed to approximate a Gaussian distribution using inverse quantile normalization. Based on these adjusted cytokine responses, we conducted 11 univariate (single-trait, single-variant) and 11 multivariate (multi-trait, single-variant) genome-wide association analyses.

In the univariate analysis, association testing was performed using linear regression implemented in PLINK2. For the multi-trait analysis, we used the MV-PLINK software, as individual-level genotype and phenotype data were available. For each genetic marker, the software tests its association with a linear combination of the selected adjusted cytokine responses—referred to as a cytokine network—that maximizes the correlation with the given marker. The test statistic is based on Wilks’ lambda (λ = 1 − ρ²), where ρ is the canonical correlation between the genetic marker and the cytokine network. *P* values are derived by transforming Wilks’ lambda into an F-statistic approximation. A separate multivariate analysis was performed for each cytokine response. The corresponding cytokine network was defined by selecting adjusted cytokine responses with an absolute Pearson correlation greater than 0.2 with the target cytokine response (see Supplementary Table 2).

The results of both GWAS analyses were visualized using the Python-based GWASLab library. Lead SNPs were defined as the most significant variants within a ± 500 kb region around each SNP, with *p* values below the genome-wide significance threshold of ${5\times 10}^{-8}$. Regional association plots were generated with a ± 100 kb window around the lead SNP. Linkage disequilibrium was calculated based on the European population reference panel from the 1000 Genomes Project. Additionally, Manhattan and quantile-quantile (QQ) plots for each cytokine response are provided in the Supplementary Figs. 5 and 6, respectively.

### Predictive modeling framework within GEOCODE

Internal predictive performance within the GEOCODE cohort was assessed using a five-fold cross-validation scheme. For each cytokine response, the GEOCODE samples were randomly and equally partitioned into five subsets, with each serving as a test set in one iteration while the remaining four subsets formed the training set. Prediction performance was evaluated by computing Spearman’s correlation between the predicted and observed cytokine levels across the 5 test sets. The average test-set performance of each predictive model was compared across all cytokine responses using Friedman’s test, followed by Conover post-hoc analyses with Holm correction. Pairwise statistical differences were then visualized using a critical difference diagram, as implemented in ref. ^51^.

Data preprocessing comprised normalization of cytokine responses, standardization of biological and environmental variables, and genetic feature selection. Each cytokine response was z-scored based on the training set, and the same scaling parameters were applied to the corresponding test set. The following biological and environmental variables were included as potential predictors: age, BMI, CMV serostatus, sex, seasonality of sample collection, blood immune cell counts (neutrophils, monocytes, lymphocytes, eosinophils, basophils), and circulating levels of sexual hormones (oestradiol, progesterone, and testosterone). All variables were z-scored based on the training set, and the same scaling parameters were applied to the corresponding test set in each cross-validation fold. Looking at the genetic feature selection, for each cross-validation fold, a univariate genome-wide association study (GWAS) was conducted for each cytokine response exclusively on the training set to select the most informative genetic markers, while avoiding data leakage into the test set. To reduce redundancy and retain the most strongly associated ones, we applied PLINK2’s clumping procedure. Genetic markers were selected with a primary p-value threshold of 1 x ${10}^{-5}$ and a secondary threshold of 1 x ${10}^{-2}$, a linkage disequilibrium threshold of ${r}^{2} > 0.5$, and a 250 kilobase window. All predictive models used this same set of selected genetic markers. In the context of genetic feature selection in the data-leaking scenario, univariate genome-wide association study (GWAS) was performed for each cytokine response using the entire GEOCODE cohort, meaning that the same GWAS results were used across all cross-validation folds. PLINK2’s clumping procedure was then applied as described in the Genetic feature selection subsection.

Several predictive models were applied. The C + T method consists in computing a polygenic score for each cytokine response using PLINK2’s score function, based on the GWAS summary statistics and clumping procedure performed within the training set. Practically, the method directly uses the GWAS-derived effect sizes to weight the selected genetic markers, without further model training. For learning models, an additional step of hyperparameter tuning was required, as model performance can be sensitive to these parameters. Hyperparameter tuning was carried out using a random search, exploring up to 25 randomly selected hyperparameter configurations. Each configuration was evaluated through an internal 5-fold cross-validation applied only to the training set. For a given learning model, the resulting hyperparameter configuration may thus vary across folds and cytokine responses. This was implemented using the *RandomizedSearchCV* function from scikit-learn.

Linear models included ordinary least squares, ridge, and elastic net regressions, implemented using the scikit-learn package. Ridge regression applies L2 regularization by penalizing the squared magnitude of the coefficients, controlled by the regularization parameter $\alpha$. Elastic Net combines both L1 and L2 regularization, balancing them through an additional parameter called the L1 ratio. For both ridge and Elastic Net, $\alpha$ was tuned over the set {1e–4, 1e–3, 1e–2, 0.1, 1, 10, 100, 1000}. In the case of Elastic Net, the L1 ratio was additionally varied across {0.1, 0.3, 0.5, 0.7, 0.9}.

Tree-based ensemble methods included random forest and gradient boosted trees, also implemented using the scikit-learn package. For random forest, 500 independent trees were grown. The hyperparameters included: (i) the maximum depth of trees, which varied across {2, 4, 6, 8, 12 and unlimited depth}, (ii) the number of features considered at each split, set to one-third, two-third, or all input features, (iii) the fraction of training samples used in each tree, chosen from {0.632, 0.8 and 1}. For gradient boosted trees, the hyperparameters were: (i) the number of trees, selected from {50, 100, 250}, (ii) the learning rate, varying from {0.01, 0.05, and 0. 1}, (iii) the maximum depth of trees, from {2, 4, 6}, and (iv) the number of features considered at each split, again set to one-third, two-third, or all input features.

The multilayer perceptron was implemented using the Keras library with a TensorFlow backend. The weights were optimized using the Adam Optimizer. Early stopping was applied to stop the training when overfitting was detected on a 10% validation split. The hyperparameters included: (i) the number of hidden layers {1, 2, 3}, (ii) the number of neurons per layer {20, 50,100, 200}, (iii) the batch size {1, 32, 64}, (iv) the initial learning rate {1e–4,5e–3, 1e–3}, and L1 and L2 regularization parameters {0, 1e–4, 1e–6}.

The analysis of input feature importance for predicting the cytokine responses was conducted with the *permutation_importance* function from scikit-learn. For each input feature, the importance score is computed based on the decrease in Spearman correlation between predicted and observed values of the test set, averaged over 50 different permutations. The distribution of these averaged feature importance scores across the 5 cross-validation folds is shown as boxplots.

### Predictive modeling framework for MI cohort

To validate externally the predictive performance, we trained the Random Forest model on the GEOCODE cohort and then applied it to the Milieu Interieur cohort. The Milieu Interieur cohort was restricted to non-smokers (n = 497). Prediction performance was evaluated by computing Spearman’s correlation between the predicted and measured cytokine response levels in the Milieu Interieur cohort. This validation was performed independently for each cytokine response that was measured in both cohorts.

Data preprocessing was also performed for the external Milieu Interieur cohort. Individuals with missing cytokine values were excluded, yielding final sample sizes of 479 for TCR IL-2, 476 for TCR IFN-γ, 477 for LPS IL-6, and 478 for LPS TNF-α. For visualization purposes, both the predicted and the observed cytokine responses in the Milieu Interieur cohort were log2-transformed. The following input variables, available in both cohorts, were included: body mass index, age, sex, CMV serostatus, seasonality of sample collection, and blood immune cell counts (neutrophils, lymphocytes, monocytes, basophils, and eosinophils). The variables in the GEOCODE cohort were z-scored, and the same normalization parameters (mean and standard deviation) were applied to the corresponding variables in the Milieu Interieur cohort.

Looking at the genetic feature selection, SNPs were selected using the C + T method based on genome-wide association studies conducted in the full GEOCODE cohort, with the same parameters as used in the internal validation. To ensure compatibility between cohorts, only genetic markers present in both GEOCODE and Milieu Interieur cohorts were retained. Because the GEOCODE cohort uses the GRCh38 reference genome and the MI cohort uses GRCh37, a liftover of SNP positions from GRCh37 to GRCh38 was performed using the CrossMap tool. The final list of selected genetic markers is provided in Supplementary Table 4.

Predictive models were based on random forest models, trained using the same hyperparameter search space as in the internal validation within the GEOCODE cohort. For TCR IL-2, the model used a maximum tree depth of 8, with one-third of the features considered at each split and 63.2% of the training samples used per tree. For TCR IFN-γ, the model used fully expanded trees (i.e., no depth limit), with one-third of the features considered and 80% of the training samples per tree. For TLR4 IL-6, the best parameters included a maximum depth of 12, two-thirds of the features considered at each split, and 63.2% of the samples used per tree. Finally, for TLR4 TNF-α, the model used a maximum depth of 8, considered all features at each split, and used 80% of the training samples per tree.

The feature importance analysis in the Milieu Intérieur (MI) cohort followed the same procedure used for internal validation in the GEOCODE cohort.

### PGS calculation

Polygenic scores for immune-mediated diseases were calculated using the pgscatalog/pgsc_calc pipeline, developed as part of the Polygenic Score Catalog^33^. The selected scores were applied to genotype data from both the GEOCODE and Milieu Interieur (MI) cohorts, as listed in Supplementary Table 5. Note that genotype data were aligned to the appropriate genome build (GRCh38 for GEOCODE and GRCh37 for MI).

### Association between PGS and cytokine responses

We follow the approach of Baker et al.^24^ to evaluate the associations between polygenic scores (PGSs) and cytokine responses. For each PGS profile, the Spearman correlation was calculated with each cytokine response measure. To evaluate statistical significance, a permutation-based method was employed: the cytokine response values were randomly permuted 1000 times, and a correlation was computed for each permuted dataset. An observed correlation was considered statistically significant if it occurred in less than 5% of the correlations obtained from the permuted null distribution (*p* < 0.05).

## Supplementary information

Supplementary Materials

## Data Availability

Data can be found at https://ega-archive.org/datasets/EGAD50000001320.
