# Supplementary material for: A machine learning framework reveals key drivers of cytokine responses in a healthy human cohort
Source: NPJ Syst Biol Appl. 2026 Feb 24;12:45. doi: 10.1038/s41540-026-00671-w (PMC13043660; doi:10.1038/s41540-026-00671-w)
Supplement: Supplementary file 1 — Supplementary Materials [file 41540_2026_671_MOESM1_ESM.pdf]

Supplementary Figures

Supplementary Figure 1

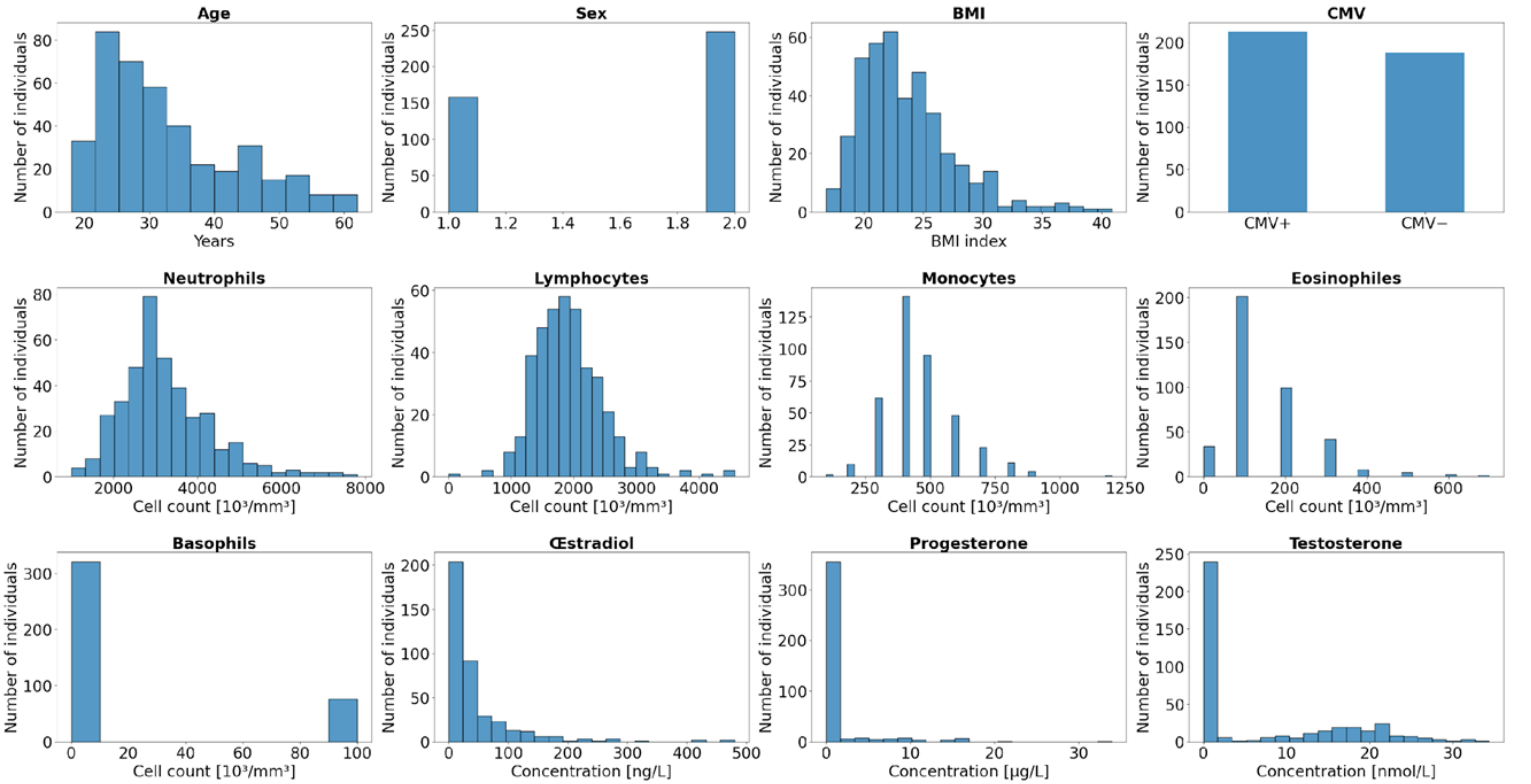

Supplementary Figure 1: Histograms of the baseline characteristics in the GEOCODE cohort.

Supplementary Figure 2

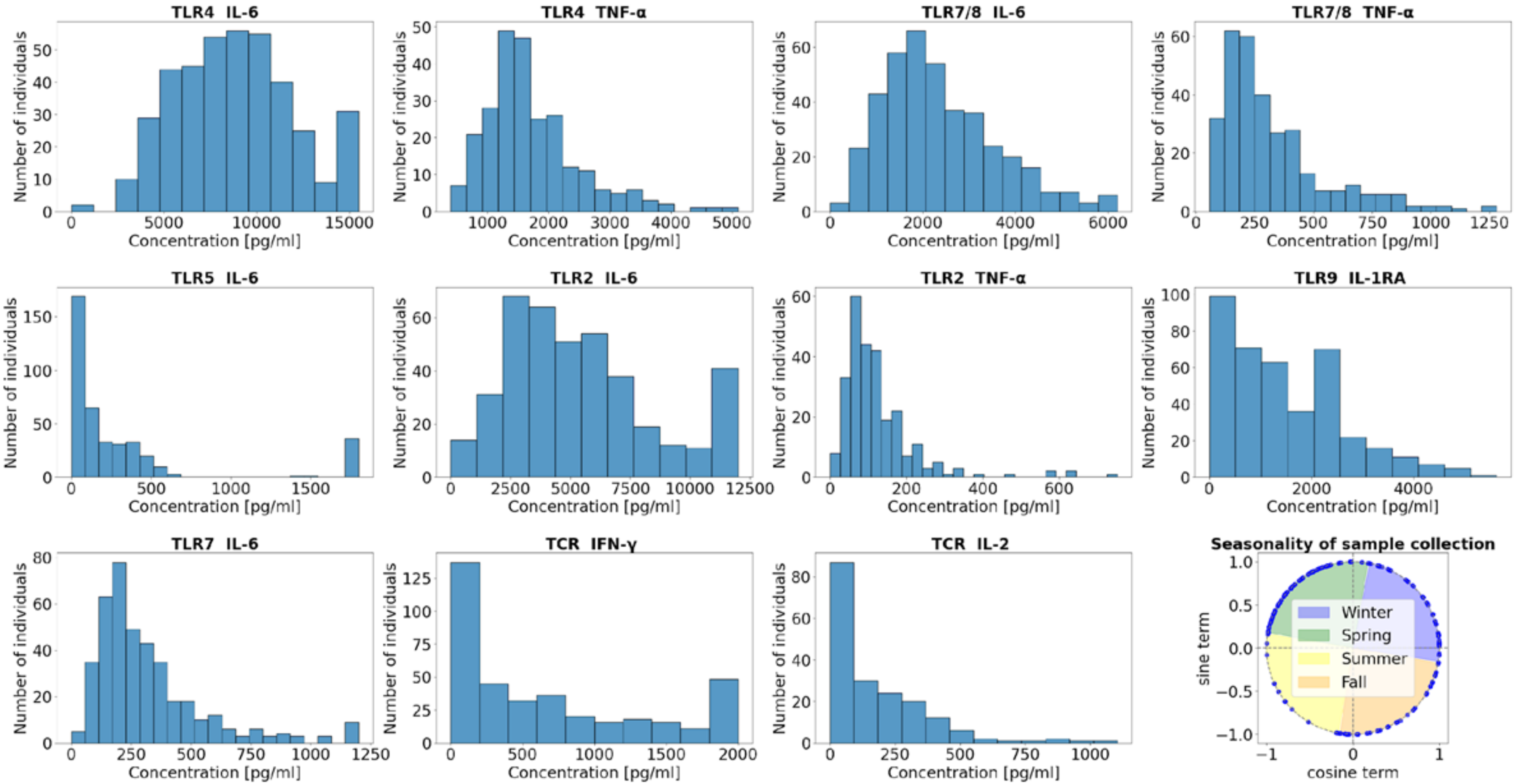

Supplementary Figure 2: Histograms of the 11 cytokine responses and the seasonality of sample collection across the GEOCODE cohort. The seasonality of sample collection is depicted by the circle, where each sample is positioned on a unit circle according to the day of the year corresponding to its collection date, using a sine and cosine transformation with a one-year period (see **Methods section seasonality modelling**)

Supplementary Figure 3

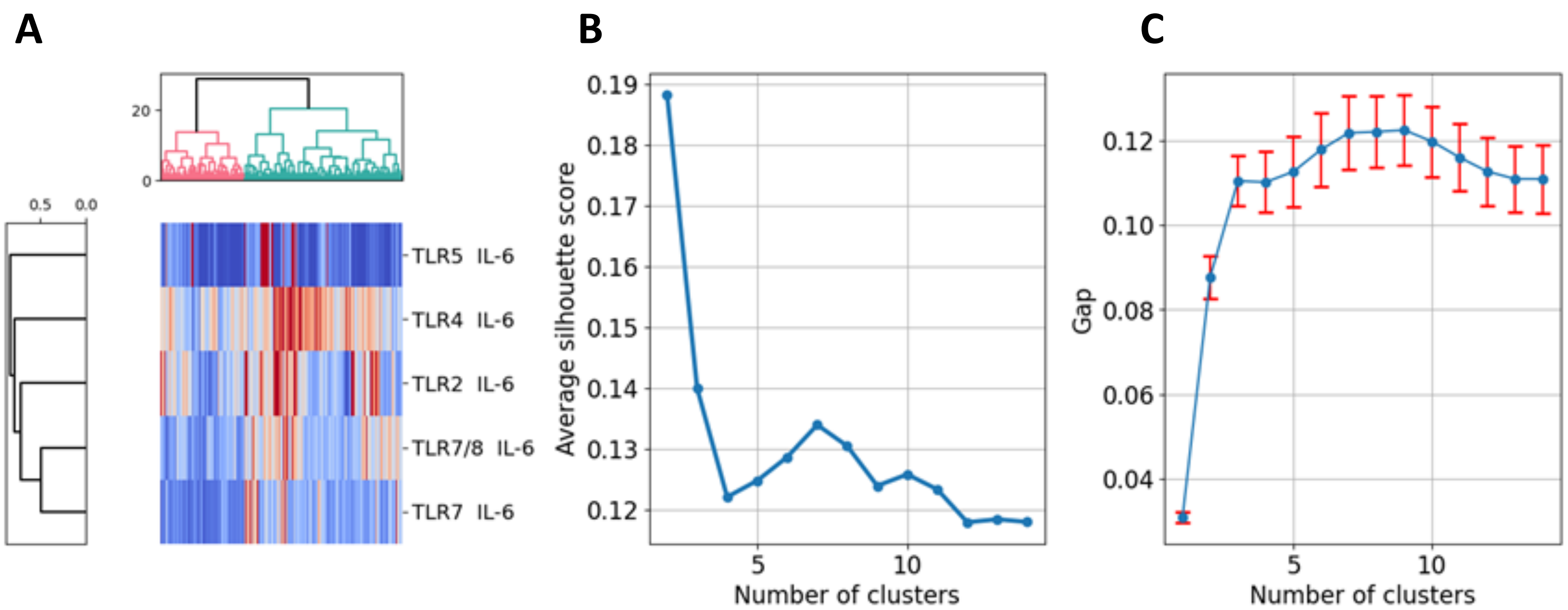

Supplementary Figure 3: Hierarchical clustering of individuals based on IL-6 cytokine response profiles and evaluation of the optimal number of clusters

A: Heatmap of IL-6 cytokine responses (rows) across individuals (columns). The color reflects cytokine response magnitude, with red indicating higher values and blue lower values. Dendrograms represent hierarchical clustering of cytokine responses (left) and individuals (top).

B: Average silhouette score for varying numbers of clusters.

C: Gap function for varying number of clusters.

Supplementary Figure 4

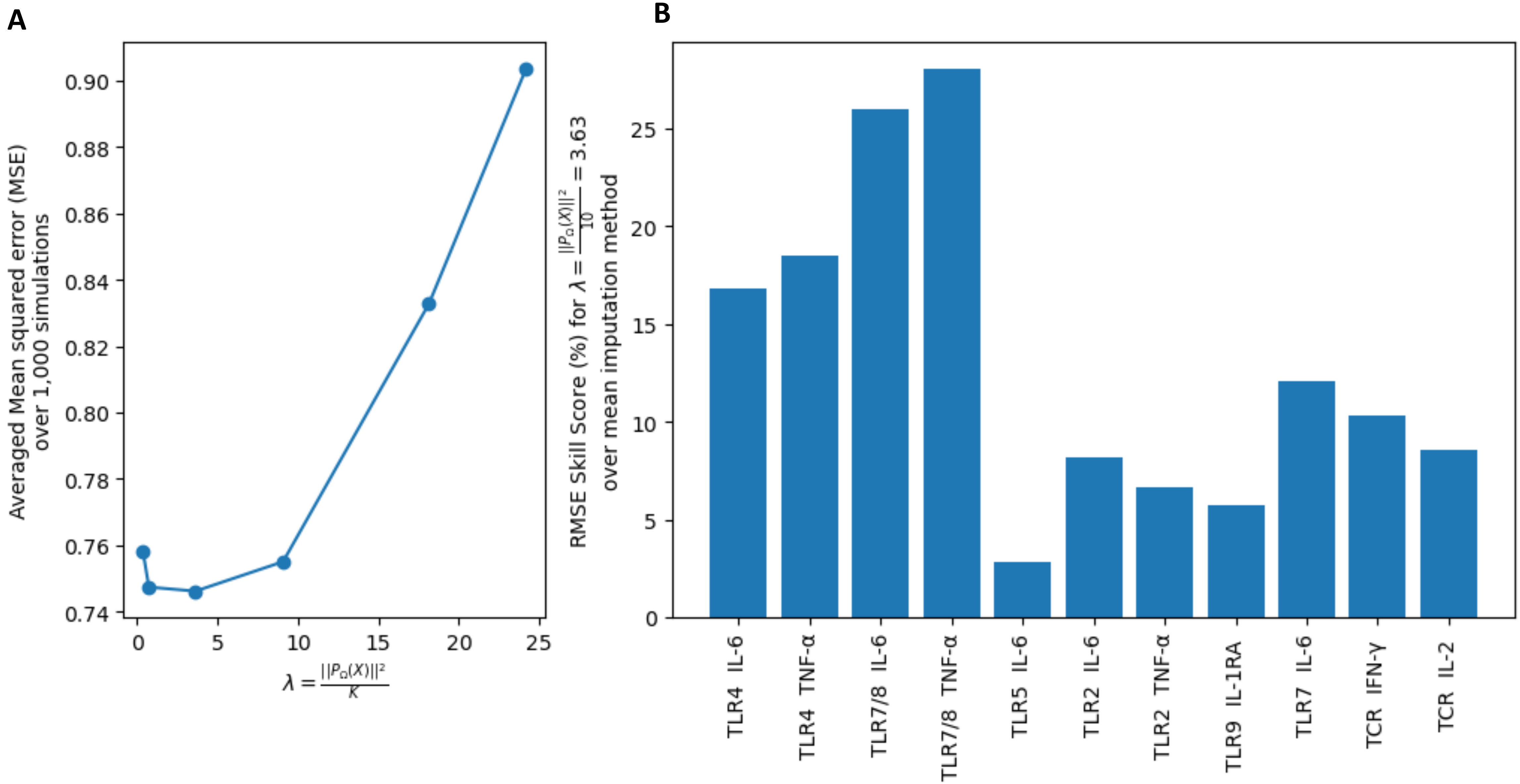

Supplementary Figure 4: Hyperparameter tuning of the SoftImpute regularization parameter (λ).

A: The average mean squared error (MSE) obtained for different values of λ in the SoftImpute algorithm, computed across 1,000 simulations in which one observed value per column was randomly masked.

B: The relative improvement in imputation accuracy compared to mean imputation, averaged over the 1,000 simulations. The improvement is expressed as a percentage using the Root Mean Square Error (RMSE) skill score for each cytokine response, calculated using the optimal λ value.

Supplementary Figure 5

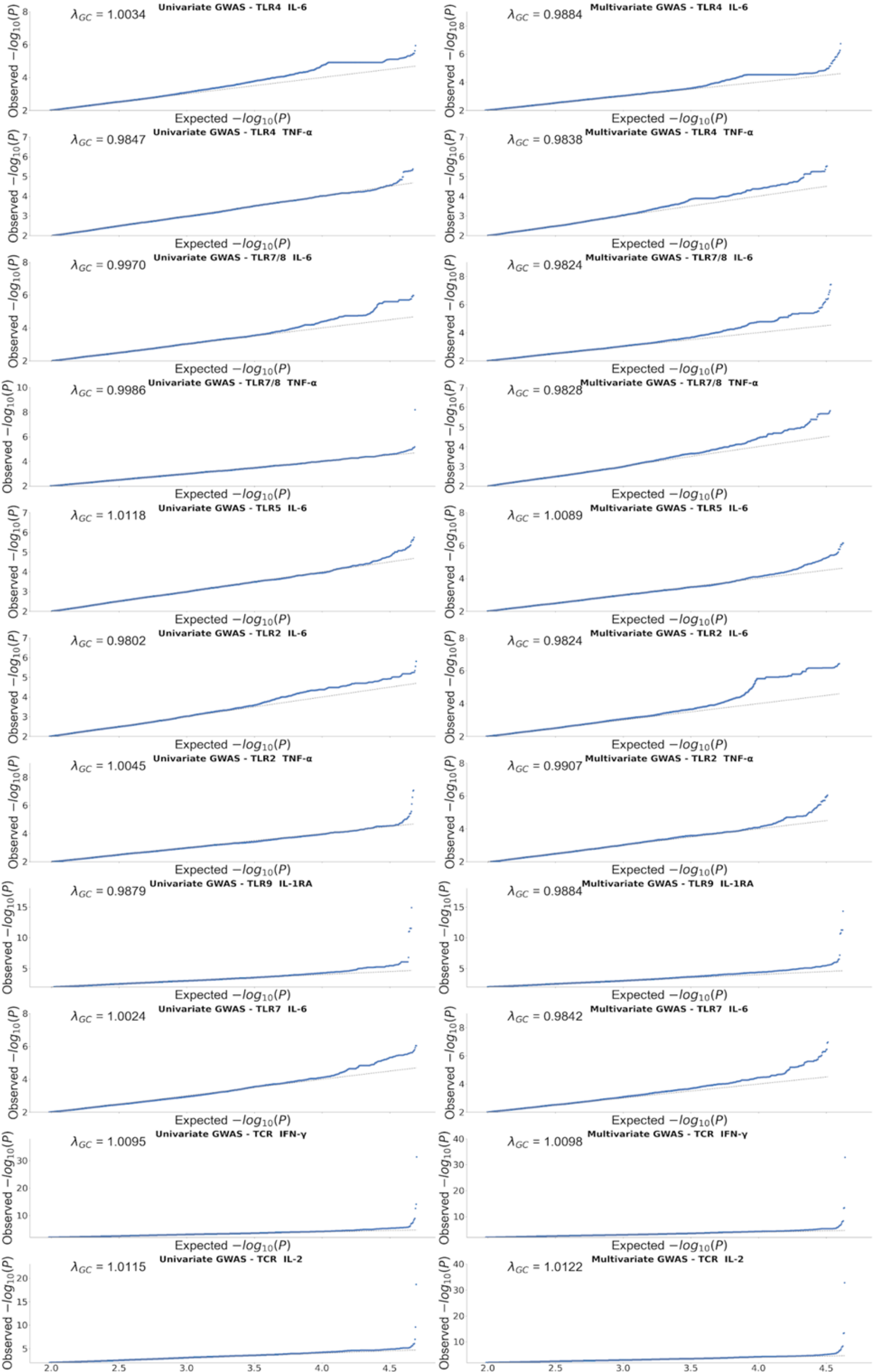

Supplementary Figure 5: Quantile-Quantile (QQ) plots

Quantile-Quantile (QQ) plots for each cytokine response from the univariate (right) and multivariate (left) GWAS analyses. Genomic inflation factor is notified by lambda (λ)

Supplementary Figure 6

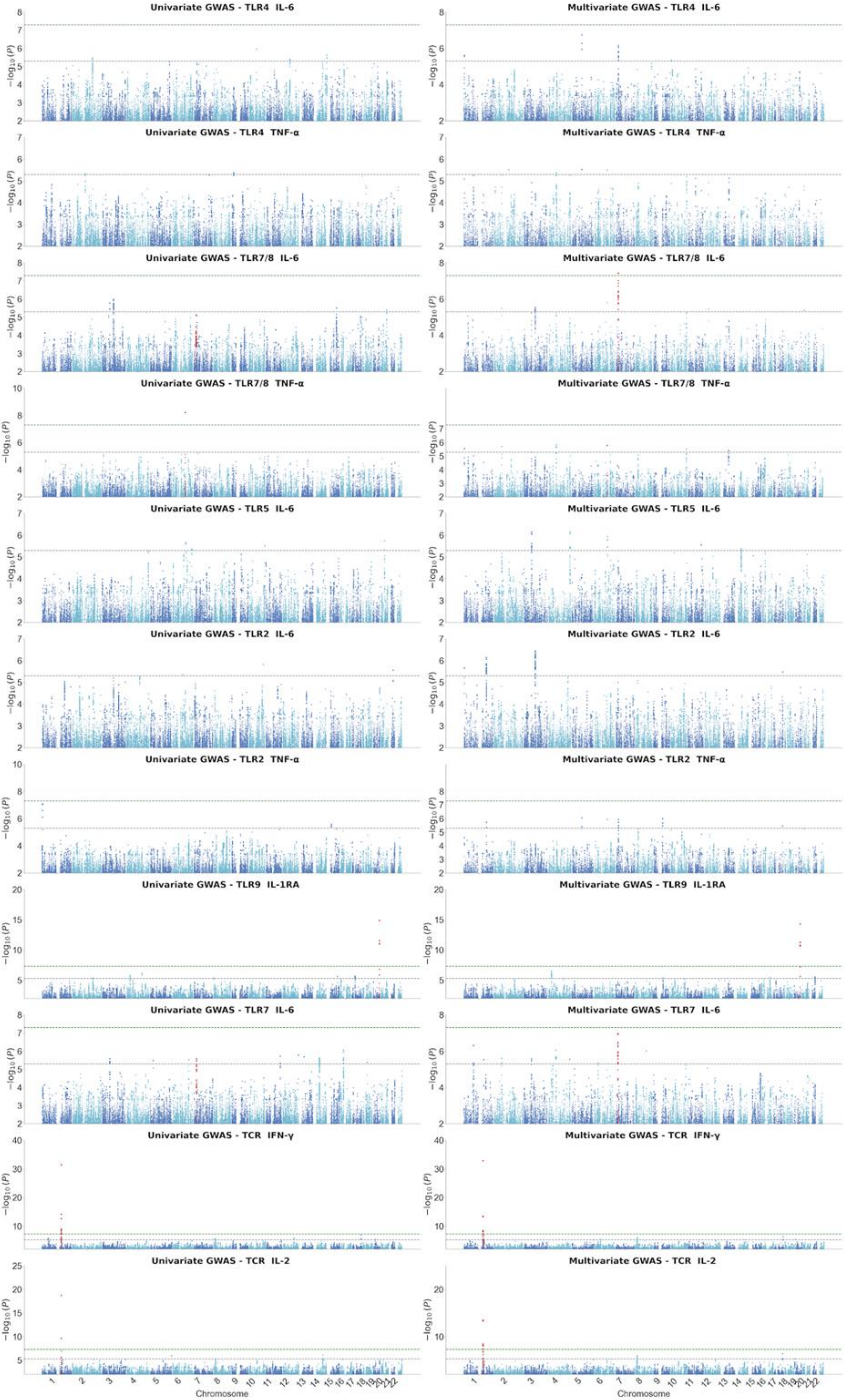

Supplementary Figure 6: Manhattan plots

Manhattan plots for each cytokine response from the univariate (right) and multivariate (left) GWAS analyses. The genome-wide significance threshold (5×10^{-8}) is indicated by a green line. Significant SNPs are highlighted in red within a 500 kb window.

Note that the genome-wide significant locus rs74513903 for TLR7 IL-6 was displayed in red, as it shows a strong loading in the multivariate analysis of IL-6 following TLR7/8 stimulation.

Supplementary Figure 7

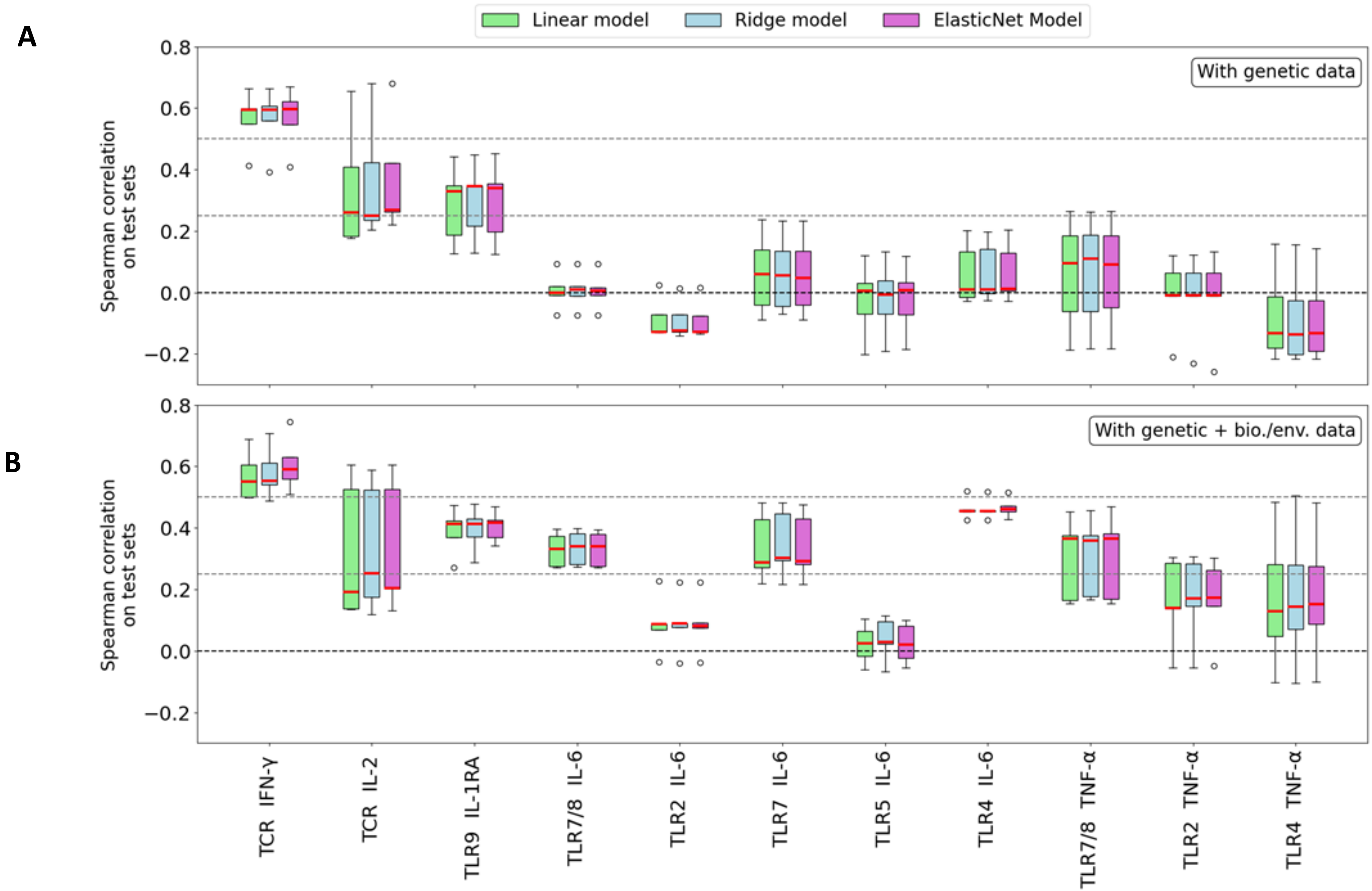

Supplementary Figure 7: Benchmark of linear models across 11 cytokine responses

A: Comparison of test performance across five-fold cross-validation for linear predictive models using only genetic data on the 11 cytokine responses. Model performance was measured by Spearman correlation.

B: Comparison of test performance across five-fold cross-validation for linear predictive models combining genetic, biological and environmental data on the 11 cytokine responses.Model performance was measured by Spearman correlation.

Supplementary Figure 8

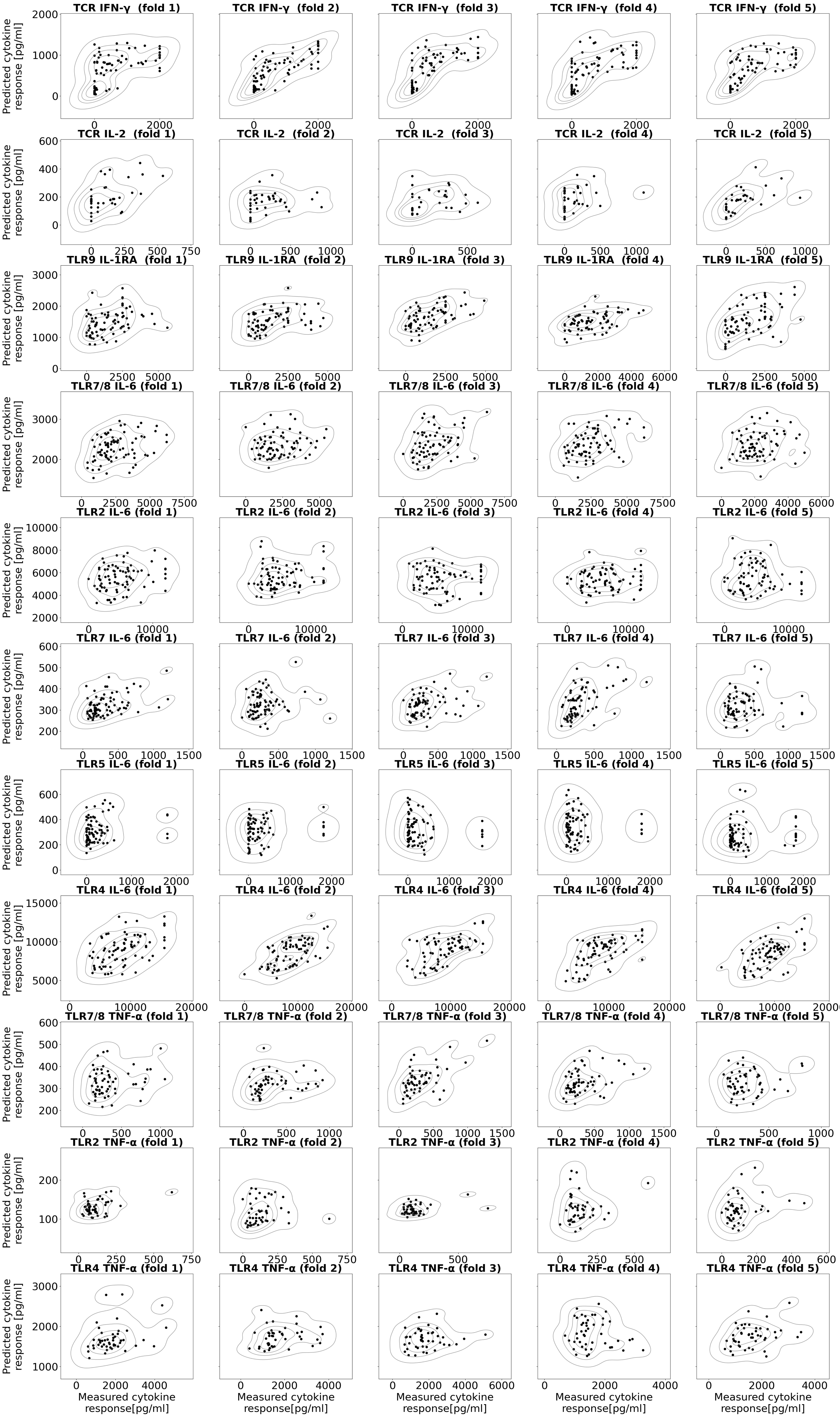

Supplementary Figure 8: Plots of cytokine response predictions developed by the random forest (RF) model.
Actual vs predicted cytokine response values across the 5 cross-validation folds for each of the 11 cytokine responses using the RF model, shown as scatter plots with density contour lines.
Each subplot shows the actual measurements (blue) with the corresponding predictions (orange) on the respective test sets.

Supplementary Figure 9

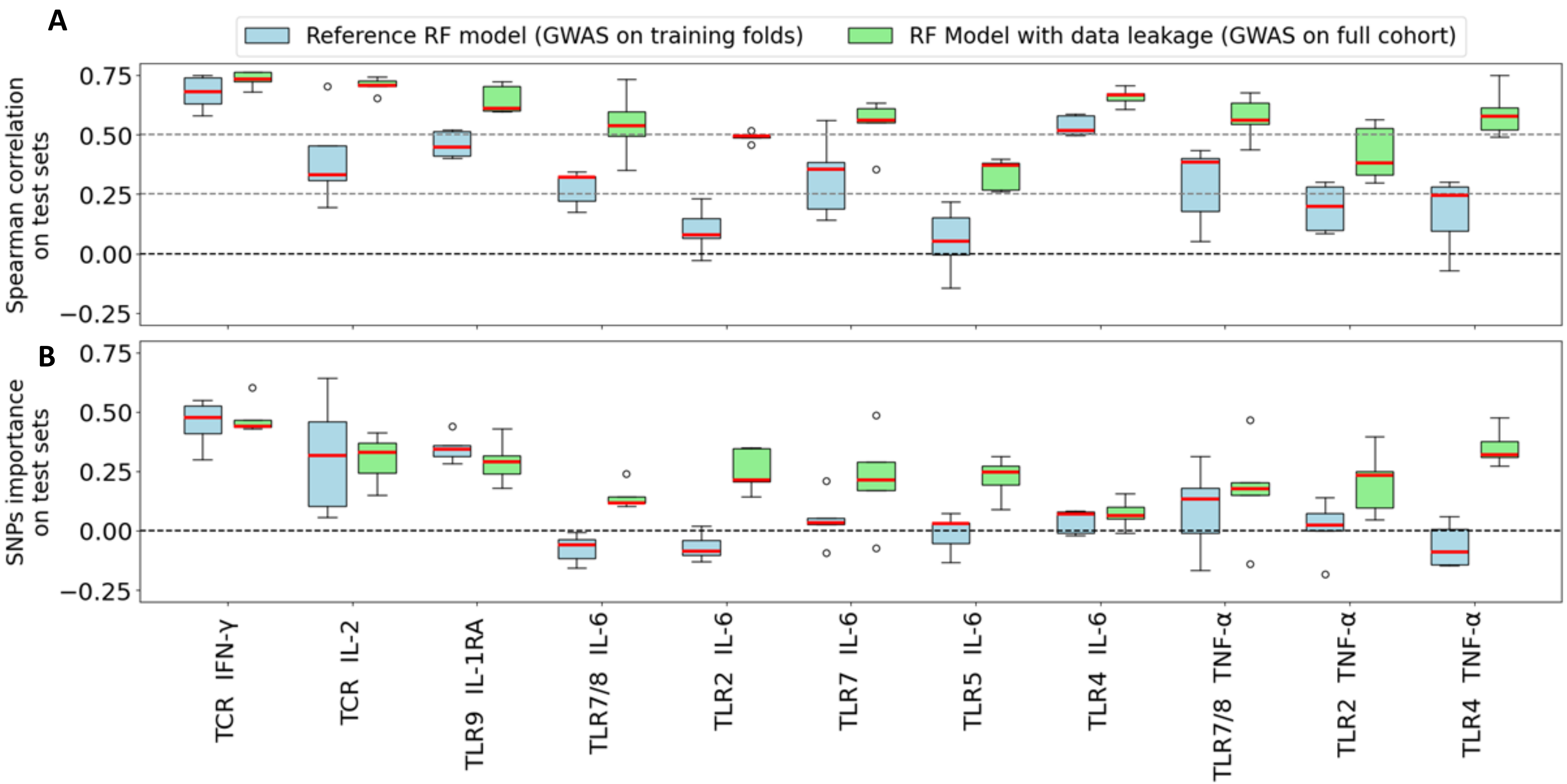

Supplementary Figure 9: Impact of data leakage in SNP selection on model performance and genetic feature importance

A: Comparison of Random Forest model performance, measured by Spearman correlation on the test sets, under two SNP selection approaches: (i) selection based on a GWAS conducted on the entire cohort (model with data leakage - green boxplots), and (ii) selection based only on the training folds within the cross-validation procedure (the reference model we used in this paper - blue boxplots).

B: Relative importance of genetic features in both scenarios, estimated by the decrease in predictive model performance (Spearman correlation) when input variables are randomly permuted.

Supplementary Figure 10

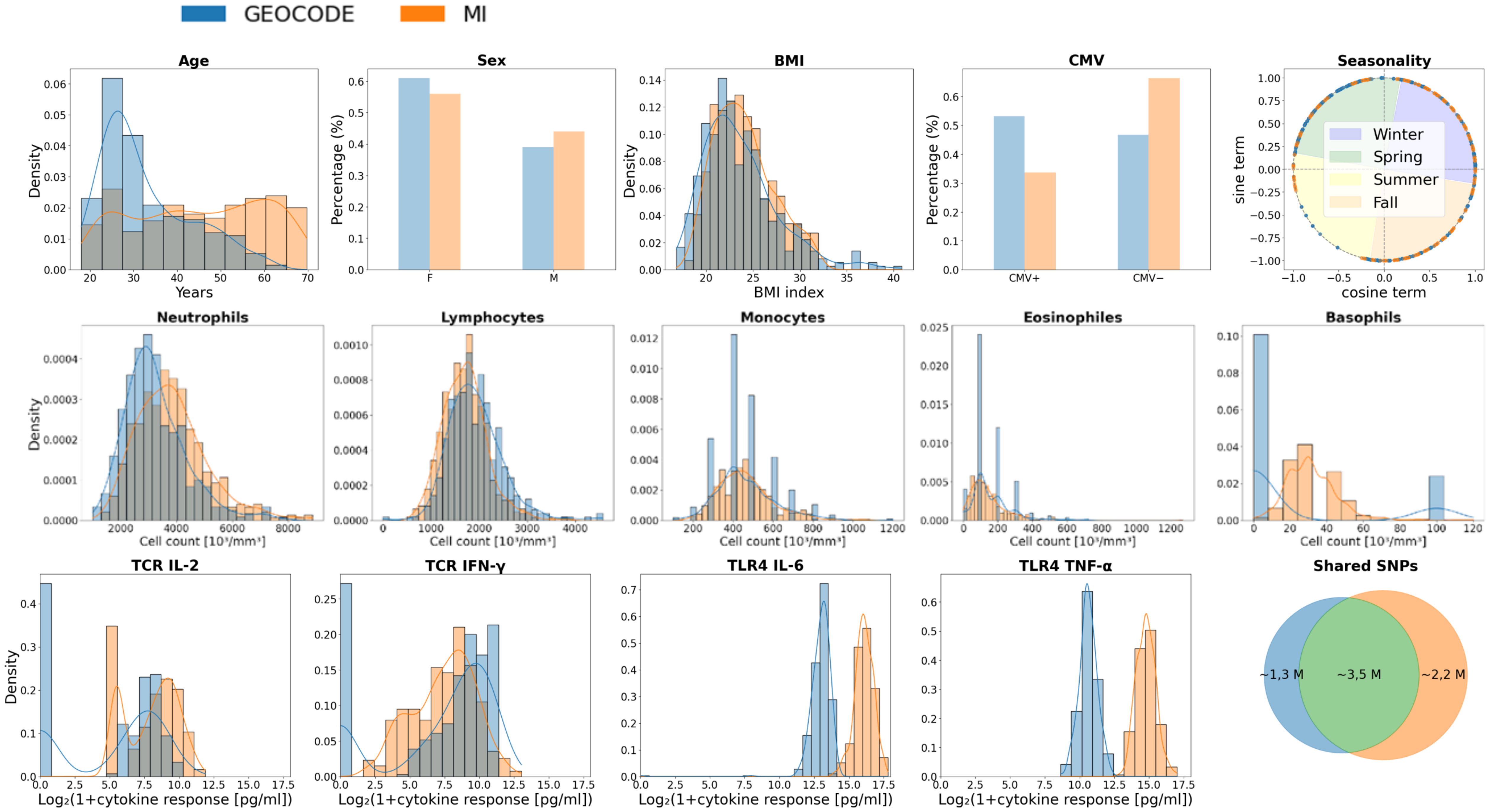

Supplementary Figure 10: Comparison between Geocode and Milieu Intérieur (MI) cohorts

Shared baseline covariates, cytokine responses following 24h of stimulation, season of sampling and genetic variants between non-smokers from MI cohort and participants from the GEOCODE cohort.

Supplementary Figure 11

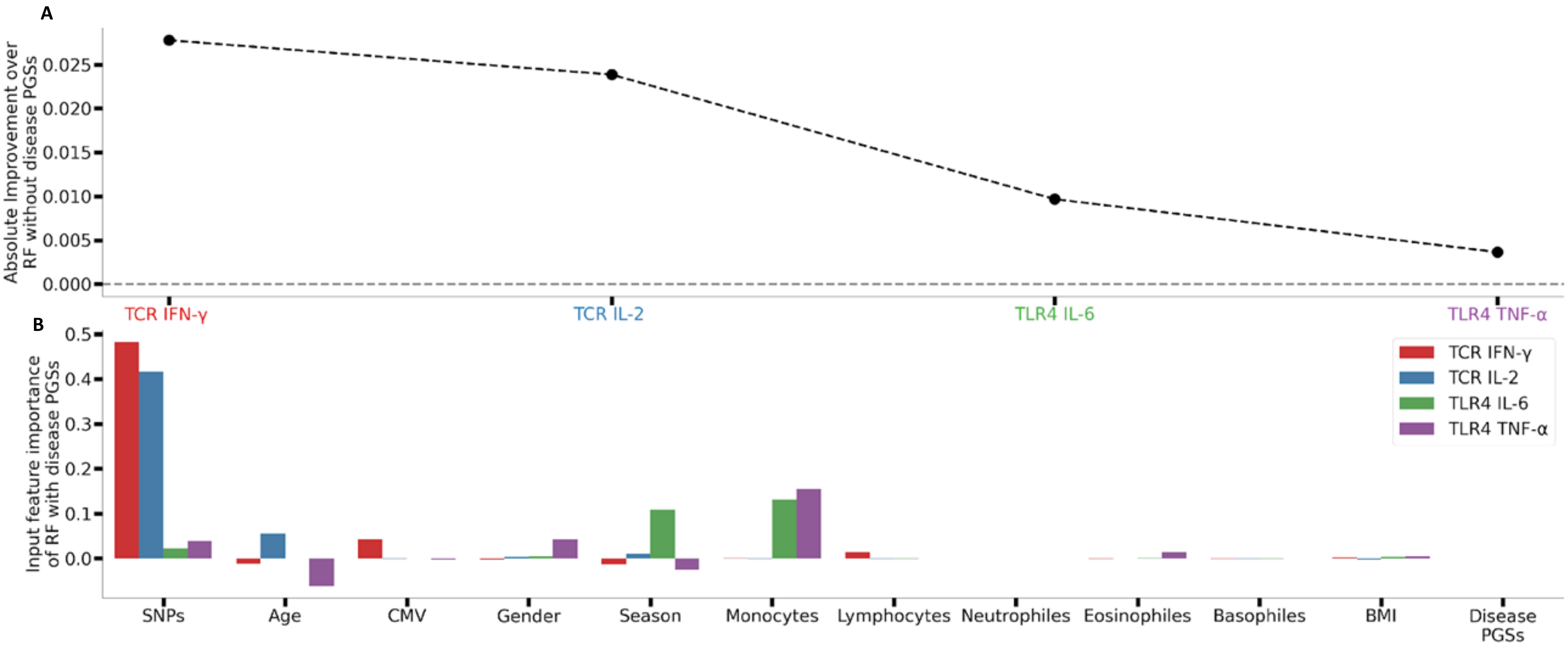

Supplementary Figure 11: Impact of autoimmune disease polygenic scores (PGSs) on Random Forest (RF) model performance and feature importance.

A: Absolute gain in predictive performance of the RF model when including the seven autoimmune disease PGSs (inflammatory bowel disease, with ulcerative colitis and Crohn's disease, multiple sclerosis, psoriasis, type 1 diabetes, and rheumatoid arthritis), compared to the same model without them. The model was trained on the GEOCODE cohort and tested on the Milieu Intérieur (MI) cohort.

B: Input feature importance of the RF model evaluated on the MI cohort. Importance was evaluated as the decrease in Spearman correlation after random permutation of each input variable

Supplementary tables

Supplementary Table 1

Covariates	Test statistic	Statistic	p-value	Bonferroni corrected p-value
CMV	Chi-square test	0	1	1
Gender	Chi-square test	22,12779526	2,55089E-06	3,57125E-05
Age	Mann-Whitney U test	19695	0,121191323	1
BMI	Mann-Whitney U test	17405	0,6245875	1
cos(doy)	Mann-Whitney U test	11993	3,08141E-08	4,31398E-07
sin(doy)	Mann-Whitney U test	15318,5	0,012189391	0,170651469
Basophiles	Mann-Whitney U test	17413	0,98511022	1
Neutrophile	Mann-Whitney U test	17074,5	0,953820002	1
Lymphocytes	Mann-Whitney U test	14404,5	0,004982374	0,069753236
Eosinophiles	Mann-Whitney U test	15840,5	0,203147378	1
Monocytes	Mann-Whitney U test	11125,5	1,22647E-09	1,71706E-08
Œstradiol	Mann-Whitney U test	18211	0,408884135	1
Progesterone	Mann-Whitney U test	17999,5	0,516067467	1
Testosterone	Mann-Whitney U test	11597	3,70622E-08	5,1887E-07

Supplementary Table 2

Central cytokine response	Corresponding cytokine networks
TCR IFN-γ	TCR IFN-γ,TCR IL-2
TCR IL-2	TCR IFN-γ,TCR IL-2
TLR9 IL-1RA	TLR9 IL-1RA,TLR7 IL-6
TLR5 IL-6	TLR5 IL-6,TLR7 IL-6
TLR4 TNF-α	TLR4 IL-6, TLR4 TNF-α, TLR7/8 IL-6, TLR7/8 TNF-α, TLR2 TNF-α, TLR7 IL-6
TLR7 IL-6	TLR4 TNF-α,TLR7/8 IL-6,TLR7/8 TNF-α,TLR5 IL-6,TLR9 IL-1RA,TLR7 IL-6
TLR7/8 IL-6	TLR4 TNF-α, TLR7/8 IL-6, TLR7/8 TNF-α, TLR2 IL-6, TLR7 IL-6
TLR7/8 TNF-α	TLR4 TNF-α, TLR7/8 IL-6, TLR7/8 TNF-α, TLR2 TNF-α, TLR7 IL-6
TLR4 IL-6	TLR4 IL-6, TLR4 TNF-α, TLR2 TNF-α
TLR2 IL-6	TLR7/8 IL-6, TLR2 IL-6, TLR2 TNF-α
TLR2 TNF-α	TLR4 IL-6,TLR4 TNF-α,TLR7/8 TNF-α,TLR2 IL-6,TLR2 TNF-α

Pearson correlation matrix of the imputed and adjusted cytokine response levels

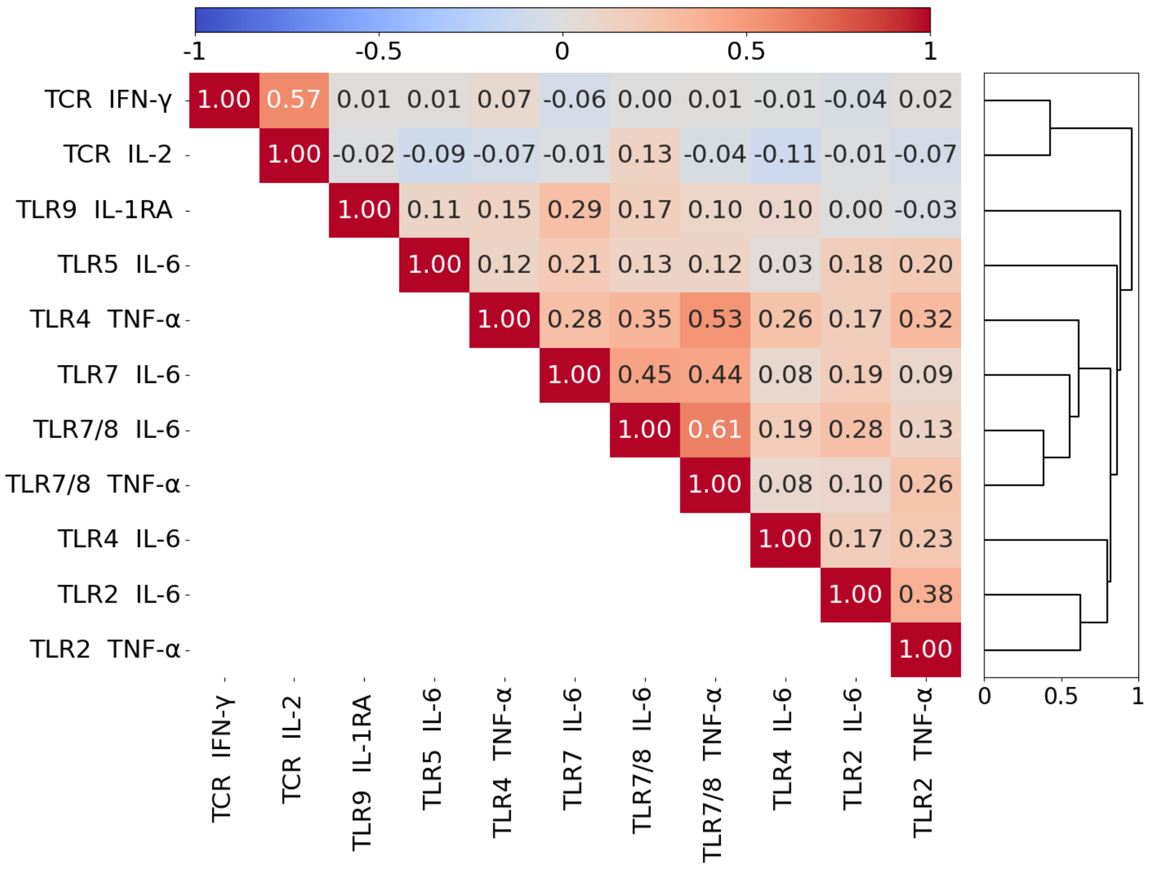

Supplementary Table 3

**Multivariate association with rs35345753 using MV-PLINK**	
**Cytokine network centered on TLR7/8 IL-6**	**Loadings**
TLR7 IL-6	0.7234
TLR7/8 IL-6	0.621
TLR2 IL-6	0.2278
TLR4 TNF-α	-0.06992
TLR7/8 TNF-α	0.02395
p-value of 3.75×10^{-8}	

Supplementary Table 4

List of genetic markers selected for external validation in the Milieu Intérieur cohort.												
	**TCR IL-2**			**TCR IFN-γ**			**LPS IL-6**			**LPS TNF-α**		
	**Pos (GR38)**	**Ref. Allele**	**Alt. Allele**	**Pos (GR38)**	**Ref. Allele**	**Alt. Allele**	**Pos (GR38)**	**Ref. Allele**	**Alt. Allele**	**Pos (GR38)**	**Ref. Allele**	**Alt. Allele**
0	chr1:161502368	A	G	chr1:51899674	A	C	chr1:221726013	G	A	chr5:6254723	A	G
1	chr1:161509955	A	G	chr1:161434464	G	A	chr2:167090575	T	C	chr9:10270853	A	AT
2	chr2:60672327	T	C	chr1:161473308	T	G	chr7:22704823	A	T			
3	chr2:164779822	T	C	chr1:161486396	G	T	chr10:59952908	A	G			
4	chr6:132678304	C	T	chr1:161502368	A	G	chr12:55541245	G	A			
5	chr6:132680842	C	T	chr1:161503335	G	C	chr14:67740174	T	G			
6	chr8:37680504	C	T	chr1:161509955	A	G	chr14:103206393	C	A			
7	chr8:125619384	A	G	chr1:161522797	C	T						
8	chr10:3945954	T	G	chr8:15583760	T	A						
9	chr11:119859406	T	C	chr11:128609616	T	C						
10	chr15:95608685	C	T	chr12:92174057	T	C						
11	chr16:6464048	C	T									

Supplementary Table 5

Auto-immune diseases	Polygenic score ID in PGS Catalog	Source Article for PGS	Number of variants	SNP Coverage in GEOCODE (%)	SNP Coverage in Milieu Intérieur (%)
IBD	PGS004038	Monti R et al. Am J Hum Genet (2024)	1018068	79.5	88.4
UC	PGS004253	Middha P et al. Nat Commun (2024)	744574	80.9	87.7
CD	PGS004254	Middha P et al. Nat Commun (2024)	744681	80.9	87.7
Psoriasis	PGS000244	Folkersen L et al. Nat Metab (2020)	552236	89.3	94.4
MS	PGS001270	Tanigawa Y et al. PLoS Genet (2022)	41	61	75.6
RA	PGS004049	Monti R et al. Am J Hum Genet (2024)	373627	84	93.7
T1D	PGS004035	Monti R et al. Am J Hum Genet (2024)	56562	82.6	89.9
